# IRAP Drives Ribosomal Degradation to Refuel Energy for Platelet Activation during Septic Thrombosis

**DOI:** 10.1002/advs.202411914

**Published:** 2025-01-24

**Authors:** Baichuan Xu, Xianpeng Ye, Kangfu Sun, Liang Chen, Zhaoyang Wen, Qigang Lan, Jun Chen, Mo Chen, Mingqiang Shen, Song Wang, Yang Xu, Xi Zhang, Jinghong Zhao, Junping Wang, Shilei Chen

**Affiliations:** ^1^ State Key Laboratory of Trauma and Chemical Poisoning Institute of Combined Injury Chongqing Engineering Research Center for Nanomedicine College of Preventive Medicine Army Medical University (Third Military Medical University) Chongqing 400038 China; ^2^ Department of Nephrology Chongqing Key Laboratory of Prevention and Treatment of Kidney Disease Chongqing Clinical Research Center of Kidney and Urology Diseases Xinqiao Hospital Army Medical University (Third Military Medical University) Chongqing 400037 China; ^3^ Medical Center of Hematology Xinqiao Hospital State Key Laboratory of Trauma and Chemical Poisoning Army Medical University (Third Military Medical University) Chongqing 400037 China

**Keywords:** platelet, IRAP (LNPEP), ribophagy, ribosome, sepsis, thrombosis

## Abstract

Platelets play crucial roles in multiple pathophysiological processes after energy‐dependent activation. It is puzzling how such a small cellular debris has abundant energy supply. In this study, it is shown that insulin‐regulated aminopeptidase (IRAP), a type II transmembrane protein, is a key regulator for platelet activation by promoting energy regeneration during septic thrombosis. Through interaction with certain endosome membrane proteins, IRAP can not only promote granule release, but also facilitate lysosomal degradation of theoretically discarded ribosomes in an mTORC1‐ and S‐acylation‐dependent manner in activated platelets. Plentiful amino acids obtained from IRAP‐mediated ribophagy are recruited to aerobic glycolysis and then promote energy metabolism reprogramming, thereby producing abundant energy for platelet life extension and prolonged activation. Consequently, targeted blocking IRAP can dramatically alleviate platelet hyperactivation and septic thrombosis.

## Graphical abstract

In septic platelets, IRAP adsorbs ribosomal proteins (r‐proteins) and cooperates with autophagosomal proteins to promote lysosomal degradation of r‐proteins, thereby providing amino acids (AAs) required for durative glycolysis, which ensures mitochondrial function and finally provides abundant ATP for prolonged platelet activation. In addition, IRAP is also involved in SNARE complex assembly to facilitate granules release.

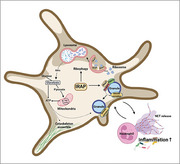

## 1 Introduction

At present, sepsis represents a major global health problem, characterized by high mortality due to a dysregulated host response to infection.^[^ ^1^, ^2^ ^]^ Thrombosis and coagulopathy, triggered by platelet hyperactivation, are leading causes of high mortality in patients with sepsis, attributing to the resultant acute organ injury and multiple organ failure.^[^ ^3^, ^4^ ^]^ However, the pathogenesis of platelet hyperactivation during sepsis is not fully understood.

Platelets, originating from the maturation of megakaryocytes, play a crucial role in maintaining homeostasis of the coagulation system. Beyond their roles in hemostasis, platelets also play a role in innate immune defense against infection and contribute to inflammation.^[^ ^5^, ^6^, ^7^ ^]^ As the main blood cells, activated platelets have unique functions that play important roles in multiple pathophysiological processes, including aggregation, granule release, and secretion of proinflammatory cytokines.^[^ ^8^, ^9^, ^10^, ^11^ ^]^ However, despite their essential roles, the intrinsic mechanisms underlying the activities of platelets remain poorly recognized. In addition to the unclarified metabolic characteristics, the significance of retaining ribosomes in anucleate platelets, which lack transcriptional requirements, remains unclear.

Platelet activation is categorically energy‐intensive.^[^ ^12^ ^]^ Initial platelet activation primarily relies on the energy generated from the mitochondrial tricarboxylic acid (TCA) cycle, whereas aerobic glycolysis emerges as the predominant supplier of energy for subsequent platelet activation.^[^ ^13^, ^14^ ^]^ However, the internal mechanism underlying this reprogramming of energy metabolism during platelet activation remains unclear. Moreover, it is elusive how such small anucleate platelets maintain a continuous supply of energy to sustain intense and persistent activation during the process of thrombosis.

Insulin‐responsive aminopeptidase (IRAP) is a type II transmembrane protein involved in endosome trafficking and immune cell responses.^[^ ^15^, ^16^, ^17^ ^]^ Variants in the IRAP (*LNPEP*) gene are associated with ischemic stroke and septic shock.^[^ ^18^, ^19^ ^]^ IRAP, in addition to its aminopeptidase function, can also bind to cytoskeletal and membrane proteins at its N‐terminal, contributing to the transport and degradation of intracellular endosomes. This characteristic has been observed in various immune cells, where it is involved in functions such as granule release, endosomal autophagy degradation, and signal transduction. Although platelets are abundant in recycling endosomes^[^ ^20^, ^21^ ^]^ the function of IRAP in platelets remains to be clarified. Therefore, the present study aimed to reveal whether IRAP can promote granule release and facilitate lysosomal degradation of the retained ribosomes to replenish energy levels required for the persistent activation of platelets during septic thrombosis.

## 2 Results

### 2.1 IRAP Intrinsically Functions in Platelet Mediated‐Septic Thrombosis

**Figure 1:**
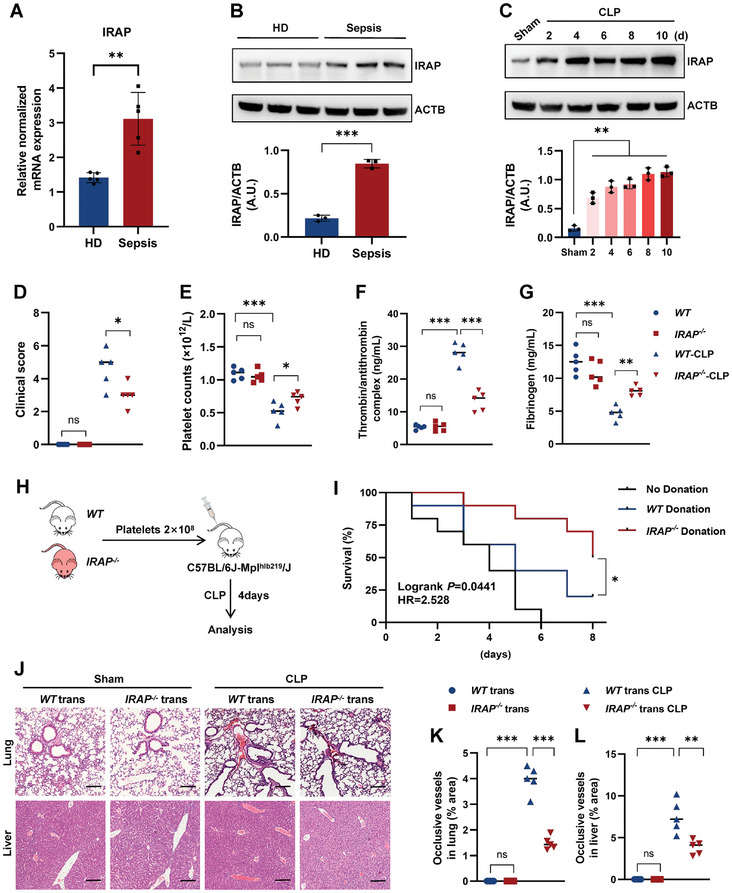
Platelet IRAP deficiency alleviates sepsis‐induced thrombosis. A) *IRAP* mRNA expression (fold change normalized to Gapdh) in platelets from healthy donors and septic patients. Each dot represents an individual experiment. B) Representative immunoblot of IRAP and β‐actin (ACTB) expression in platelets isolated from healthy donors and nonviral sepsis patients. C) Representative immunoblot of IRAP and ACTB expression in platelets isolated from wild‐type (*WT*) mice and CLP‐treated mice for 2, 4, 6, 8, and 10 days. D) Clinical score of sham and CLP‐operated *WT* and *IRAP^−/−^* mice. E) Platelet counts in sham and CLP‐operated *WT* and *IRAP^−/−^* mice. F,G) Plasma thrombin/antithrombin complex (TAT) and fibrinogen levels of sham and CLP‐operated *WT* and *IRAP^−/−^* mice. H) Experimental design for analysis of thrombosis after CLP surgery following the transfusion of either *WT* and *IRAP^−/‐^* platelets into C57BL/6J‐Mpl^hlb219^/J (*Mpl^−/−^*) recipients. I) Survival curves of platelet‐transfusion mice H) after CLP surgery to induce sepsis (n = 10, 8 weeks old male). J–L) Hematoxylin and eosin staining in livers and lungs of *Mpl^−/−^* mice transfused with *WT* or *IRAP^−/‐^* platelets in the sham and CLP groups. Statistical analysis of the staining for thrombus area in livers and lungs (n = 5). Scale bars, 100 µm. *P <.05; **P <.01; ***P <.001. ns, no significance; HD, healthy donor. IFN‐γ, interferon‐γ; LPS, lipopolysaccharide; WT, wild‐type; IRAP, insulin‐regulated aminopeptidase; CLP, cecal ligation and puncture.

To explore the functions of IRAP in platelet‐mediated‐septic thrombosis, we first examined IRAP expression in platelets during the onset of sepsis. The transcription level of *IRAP* was significantly elevated in platelets obtained from patients with sepsis compared to that in platelets obtained from healthy participants (**Figure** **1**A). Consistently, we detected a remarkable increase in IRAP protein expression in platelets from patients with sepsis compared to those from healthy donors (Figures 1B and S1A, Table S1, Supporting Information). Similar results were also observed in platelets isolated from mice subjected to cecal ligation puncture (CLP), a classic model simulating the progression and complications of human sepsis,^[^ ^22^, ^23^, ^24^ ^]^ reaching a plateau on the fourth day (Figures 1C and S1B–D, Supporting Information). These results suggest that the increase in IRAP in platelets is closely related to septic thrombosis. As expected, the canonical parameters of disseminated intravascular coagulation (DIC) detected in CLP mice were alleviated in IRAP‐deficient (*IRAP^−/−^*) mice (Figures 1D–G and S1E–I, Table S2, Supporting Information).

To comprehensively evaluate the pathological function of IRAP in septic thrombosis, we further transplanted the platelets from WT or IRAP‐null mice into thrombopoietin receptor‐deficient (C57BL/6J‐Mpl^hlb219^/J, *Mpl^−/−^*) mice,^[^ ^25^ ^]^ which have ≈10% of the number of circulating platelets (Figures 1H and S1J, Supporting Information). The survival rate of CLP‐*Mpl^−/−^* mice transfused with *IRAP^−/−^*‐platelets was considerably higher than that of CLP‐*Mpl^−/−^* mice transfused with normal platelets (Figure 1I). Moreover, CLP‐*Mpl^−/−^* mice transfused with *IRAP^−/−^*‐platelets exhibited fewer postoperative DIC indicators and less thrombosis in lung and liver tissues than the control group (Figures 1J–L and S1K–M, Supporting Information). The same phenomenon was validated in a mouse model of platelet clearance (Figure S1N–P, Supporting Information). These findings reveal an essential and intrinsic role of IRAP in platelet‐mediated sepsis thrombosis.

### 2.2 Platelet Activities in Sepsis Thrombosis are Regulated by IRAP

**Figure 2:**
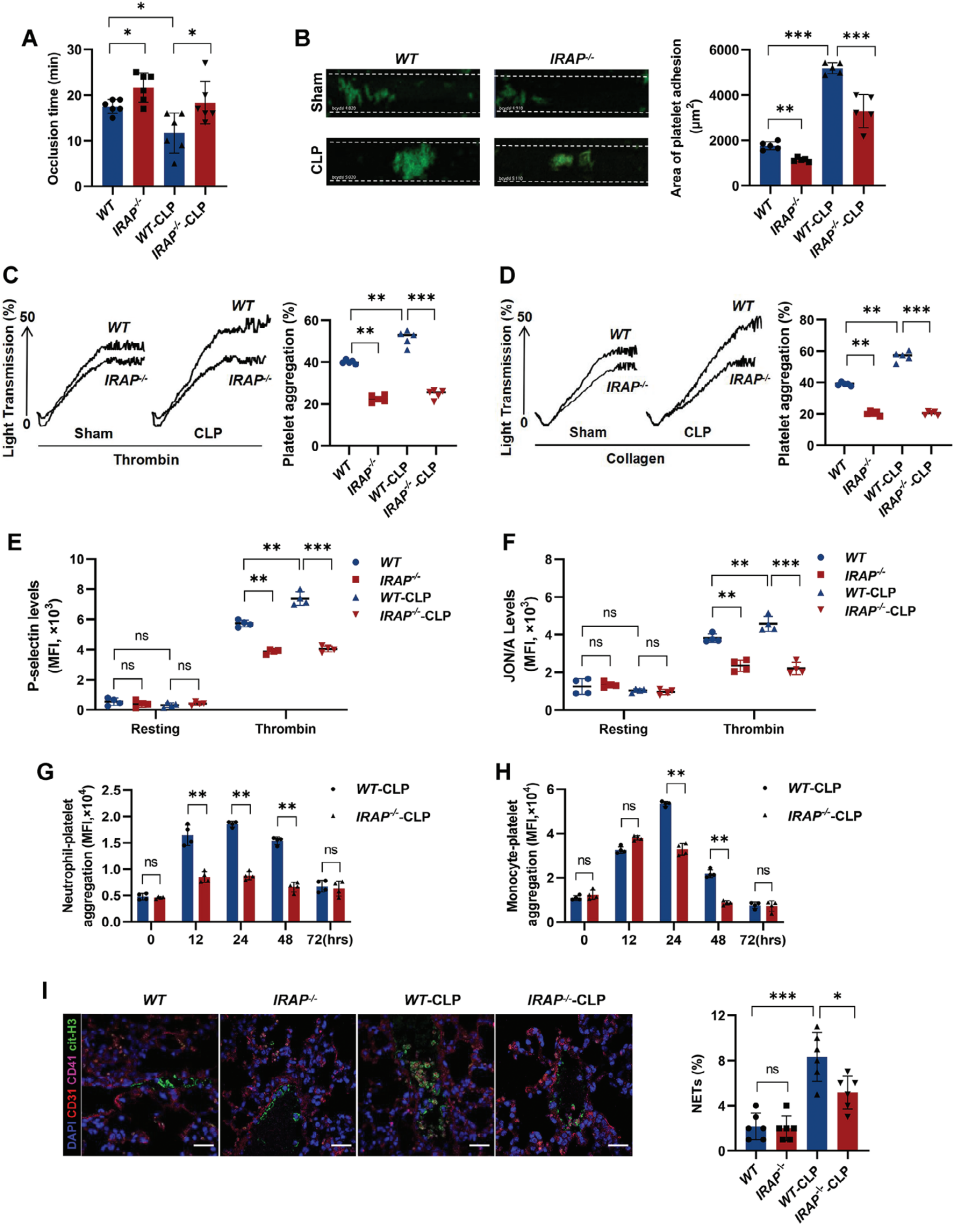
IRAP is involved in platelet activation and NET formation in sepsis. A) FeCl_3_‐induced arterial thrombosis in sham and CLP‐operated *WT* and *IRAP^−/−^* mice (n = 6). B) Platelet adhesion on collagen under flow conditions. Whole blood was labeled with mepacrine and perfused through fibrillar collagen‐coated BioFlux plates (Fluxion Biosciences) at 40 dynes cm^−2^ for 4–5 minutes. C,D) Aggregation of platelets isolated from sham and CLP‐operated *WT* and *IRAP^−/−^* mice in response to thrombin (0.01 U/mL) or collagen (0.25 µg mL^−1^) (n = 5). E,F) P‐selectin and JON/A exposure levels of platelets isolated from sham and CLP‐operated *WT* and *IRAP^−/−^* mice in response to thrombin (n = 4). G,H) Neutrophil‐platelet aggregation and monocyte‐platelet aggregation levels at the indicated time points after CLP surgery of *WT* and *IRAP^−/−^* mice (n = 5). I) Immunofluorescence images of NETs in the lung of sham and CLP‐operated *WT* and *IRAP^−/−^* mice. Platelets, endothelial cells, NETs, and nuclei were respectively stained with anti‐CD41 (purple), anti‐CD31 (red), anti‐cit‐histoneH3 (cit‐H3, green), and DAPI (blue). Scale bars, 50 µm. Quantification of cit‐H3 area per field (n = 6). *P <.05, **P <.01; ***P <.001. ns, no significance; MFI, mean fluorescence intensity; DAPI, 4′,6‐diamidino‐2‐phenylindole; hrs, hours; NETs, neutrophil extracellular traps; WT, wild‐type; IRAP, insulin‐regulated aminopeptidase; CLP, cecal ligation and puncture; ECs, endothelial cells.

To further explore the influence of IRAP on platelet function during the progression of sepsis thrombosis, we performed FeCl_3_‐induced mesenteric arterial thrombosis and tail bleeding assays. Compared with *WT* mice, *IRAP^−/−^* mice exhibited significantly extended tail bleeding time and reduced arterial thrombus formation after CLP induction (**Figures** **2**A and S2A, Supporting Information). Similarly, the ability of platelets to spread and form thrombi in vitro markedly declined in IRAP‐deficient mice during sepsis (Figures 2B and S2B, Supporting Information). IRAP‐deficient platelets presented significantly insufficient platelet aggregation compared to *WT* platelets after stimulation with collagen (0.25 µg mL^−1^) and thrombin (0.01 U mL^−1^) (Figure 2C,D). Moreover, platelets from CLP‐*IRAP^−/−^* mice showed less P‐selectin exposure, JON/A binding, and clot retraction than those from CLP‐*WT* mice (Figures 2E,F and S2C, Supporting Information). Given that P‐selectin can be de novo synthesized by platelets following integrin β3 engagement with its ligands,^[^ ^26^ ^]^ we ruled out differences in levels of total P‐selectin and integrin β3 in *WT* and *IRAP^−/−^* platelets (Figure S2D, Supporting Information).

Excessive formation of neutrophil extracellular traps (NETs) is an important cause of inflammatory thrombosis.^[^ ^27^, ^28^ ^]^ We observed that neutrophil/monocyte‐platelet aggregation, which can be induced by CLP, was significantly reduced in CLP‐*IRAP^−/−^* mice (Figure 2G,H), along with notable alleviation of neutrophil extracellular trap formation (NETosis) in the lungs, as determined using in situ immunofluorescence staining (Figure 2I). These findings demonstrate that platelet activation is regulated by IRAP in sepsis thrombosis.

### 2.3 IRAP Deficiency Impedes Granule Release and Energy Generation in Activated Platelets

**Figure 3:**
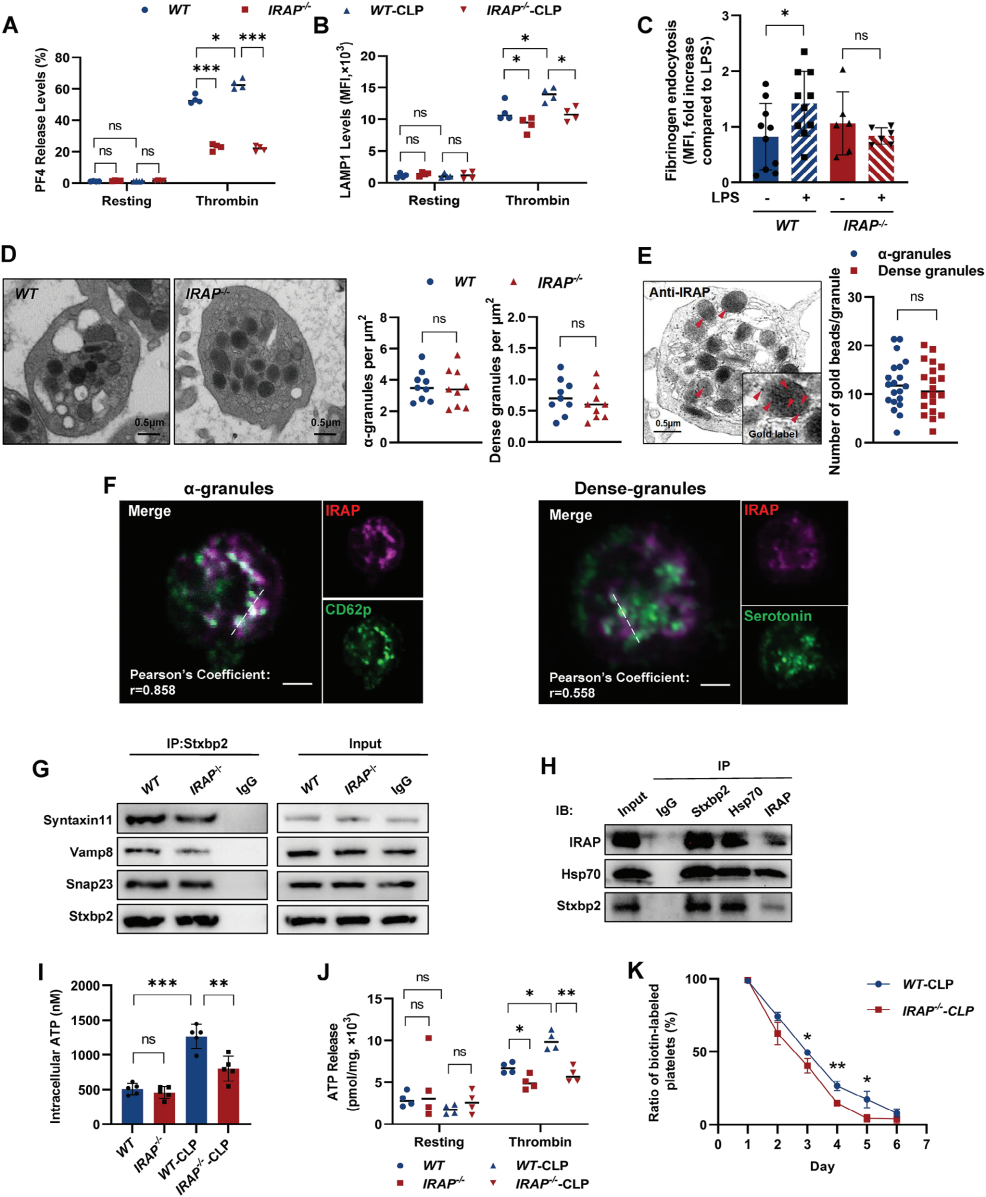
IRAP facilitates platelet granule release and ATP generation. A) PF4 levels in the supernatant of platelets isolated from sham and CLP‐operated *WT* and *IRAP^−/−^* mice in response to thrombin (n = 4). B) Flow cytometric analysis of LAMP1 in platelets isolated from sham and CLP‐operated *WT* and *IRAP^−/−^* mice in response to thrombin (n = 4). C) Platelet endocytosis experiment. Platelets were incubated with fibrinogen for 30 minutes, then washed with PBS and stained with CD41. CD41‐ and fibrinogen‐positive platelets were then measured by flow cytometry (n = 6–10). D) Representative TEM images of *WT* and *IRAP^−/‐^* platelets. Platelet granule numbers were quantified using perunit area of counted granules (n = 9). Scale bars, 500 nm. E) Representative immunoelectron microscopy (IEM) image for presenting the location of IRAP in platelet granules. Quantitative analysis of the numbers of gold beads in different platelet granules (n = 20). Scale bars, 500 nm. F) Immunofluorescence images of the co‐localization of IRAP and platelet granules in platelet. IRAP is shown in purple; platelet granules were labeled with P‐selectin (green) or Serotonin (green). Scale bars, 1µm. G) IP‐Stxbp2 assay of the lysates of platelets from *WT* and *IRAP^−/−^* mice. The SNARE complex protein expression in platelets was tested using anti‐Syntaxin11, anti‐VAMP8, and anti‐SNAP23 antibodies. H) Coimmunoprecipitation (Co‐IP) of platelet lysates with anti‐Stxbp2, anti‐Hsp70, and anti‐IRAP antibodies. Isotype‐matched IgG served as the negative control. I) Quantification of intracellular ATP in lysates of platelets isolated from *WT* and *IRAP^−/−^* mice (n = 5). J) ATP levels in the supernatant of platelets isolated from sham and CLP‐operated *WT* and *IRAP^−/−^* mice in response to thrombin (n = 4). K) Biotin‐labeled ratio of platelets at different time points after tail injection of Sulfo‐NHS‐LC‐Biotin (30 mg g^−1^ body weight) in CLP‐operated *WT* and *IRAP^−/−^* mice (n = 5, 8 weeks, male). *P <.05; **P <.01; ***P <.001. ns, not significant; MFI, mean fluorescence intensity; WT, wild‐type; IRAP, insulin‐regulated aminopeptidase; CLP, cecal ligation and puncture.

IRAP is an anchor protein for slow‐recycling endosomes.^[^ ^29^ ^]^ Given that megakaryocytes and platelets are granulosa‐rich cells, we further explored the role of IRAP in the granule release of platelets. The release of platelet granules can be detected by their respective markers. Platelet IRAP deficiency suppressed the release of various granules (**Figure** **3**A,B), suggesting that IRAP regulates granule secretion. As granule content is linked to protein uptake,^[^ ^30^, ^31^ ^]^ IRAP deletion also impaired platelet endocytosis (Figures 3C and S2E, Supporting Information). In addition, the numbers and substructures of *IRAP^−/−^* platelet granules were not altered compared to those of *WT* platelets (Figure 3D), demonstrating that IRAP deletion did not affect platelet granule numbers but inhibited their secretion. IRAP was partially located in platelet granules, further suggesting that IRAP exhibits a regulatory effect on granule secretion (Figure 3E,F).

Platelet granule release is mediated by soluble N‐ethylmaleimide‐sensitive factor attachment protein receptors (SNAREs) and their associated proteins, including syntaxin‐binding protein 2 (STXBP2), syntaxin‐11, synaptosome‐associated protein 23 (SNAP23), and vesicle‐associated membrane protein‐8 (VAMP8).^[^ ^32^, ^33^ ^]^ We found that, compared to *WT* controls, *IRAP^−/−^* platelets exhibited a reduced SNARE complex, evidenced by decreased binding of syntaxin11, VAMP8, and SNAP23 to STXBP2 (Figure 3G). Further analysis revealed that IRAP could interact with STXBP2 via HSP70 (Figures 3H and S3A, Supporting Information), indicating a structural role of IRAP in the assembly of the SNARE complex.

Notably, we observed a remarkable reduction in ATP generation and release in *IRAP^−/−^* platelets during CLP (Figure 3I,J). In addition, IRAP deficiency resulted in a short platelet lifespan during sepsis (Figure 3K). Considering that platelet survival and activation, including granule trafficking and release, are highly energy‐dependent, these results suggest that IRAP may have other unknown functions associated with energy metabolism in platelets.

### 2.4 Platelet IRAP Deficiency Leads to an Accumulation of Ribosomal Proteins

**Figure 4:**
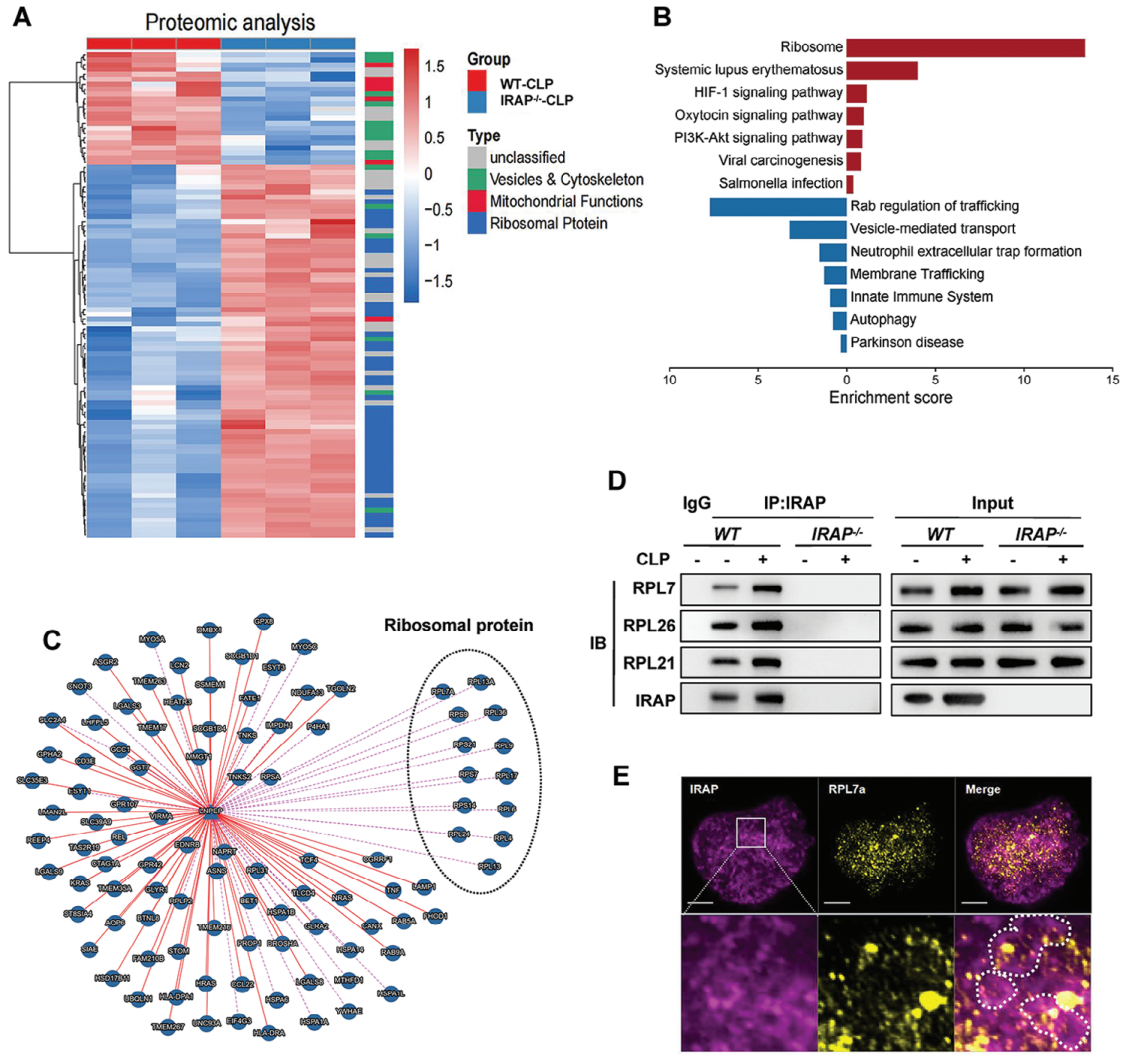
Platelet IRAP deficiency results in the accumulation of ribosomal proteins. A) Heatmap showing significant differential proteins in platelets isolated from CLP‐operated *WT* and *IRAP^−/−^* mice based on quantitative proteomic analysis. Three independent biological replicates were used for cluster analysis, with color representing the quantitative ratio. The functional classification of the protein is marked by different colors, including vesicles and cytoskeleton (green), mitochondrial function (red), and ribosomal protein (blue). B) Kyoto Encyclopedia of Genes (KEGG) of the top seven differential cell pathways (upregulation and downregulation) in CLP‐operated *WT* and *IRAP^−/−^* mice platelets based on quantitative proteomic analysis. C) Proteins that interact with IRAP were predicted using the Molecular Interaction Search Tool (MIST, https://fgrtools.hms.harvard.edu/MIST/, Harvard Medical School). Ribosomal proteins are circled by dotted lines. D) IRAP immunoprecipitation assay of the lysates of platelets isolated from sham or CLP‐operated *WT* and *IRAP^−/−^* mice. Ribosomal protein levels were tested using anti‐RPL7, anti‐RPL26, anti‐RPS15A, and anti‐RPL21 antibodies. Isotype‐matched IgG served as the negative control. E) Immunofluorescence images of the co‐localization of IRAP^+^ endosomes (purple) and RPL7a (yellow) in platelets. Scale bars, 1 µm. WT, wild‐type; IRAP, insulin‐regulated aminopeptidase; CLP, cecal ligation and puncture.

To comprehensively uncover the underlying molecular mechanisms of platelet activation impairment by the lack of IRAP, we performed quantitative proteomic analysis to screen significant changes in protein expression in CLP‐*IRAP^−/−^* platelets. The heatmap revealed three groups of differentially expressed proteins: ribosomal proteins (r‐proteins), and cytoskeletal or vesicular transport and mitochondrial proteins (**Figure** **4**A). Specifically, r‐proteins were exclusively enriched among the upregulated proteins. As presented in Table S3 (Supporting Information), 49 ribosomal subunits were upregulated, whereas none were downregulated in *IRAP^−/−^* platelets. We detected the accumulation of r‐proteins in CLP‐*IRAP^−/−^* platelets via immunoblotting (Figure S4A, Supporting Information). Furthermore, through platelet clearance and platelet transfusion, we have demonstrated that changes in ribosomal proteins content are a common effect of sepsis on both newly produced platelets and existing circulating platelets (Figure S4B,C, Supporting Information). Notably, many r‐proteins were predicted to interact with IRAP in the Molecular Interaction Search Tool database (Figure 4C). We also found that IRAP could directly interact with the representative r‐proteins via immunoprecipitation (Figure 4D). Consistent with the role of IRAP in endosome recycling, we observed that RPL7a‐labeled ribosomes were partially enveloped within IRAP^+^ endosomes in platelets from mice subjected to sepsis (Figure 4E). This result suggests that the increase in r‐proteins in platelets may be attributed to the dysfunction of protein transport. Gene Ontology (GO) and Kyoto Encyclopedia of Genes and Genomes (KEGG) pathway enrichment analyses revealed that the downregulated proteins were primarily enriched in vesicle‐mediated transport and autophagy in CLP‐*IRAP^−/−^* platelets (Figure 4B). Collectively, these results indicated that the accumulation of large amounts of r‐proteins in *IRAP^−/‐^* platelets may be ascribed to the impairment of ribosome protein degradation of platelets during sepsis.

### 2.5 Ribophagy is Responsible for the Degradation of Platelet Ribosome Protein in IRAP‐mediated Septic Thrombosis

**Figure 5:**
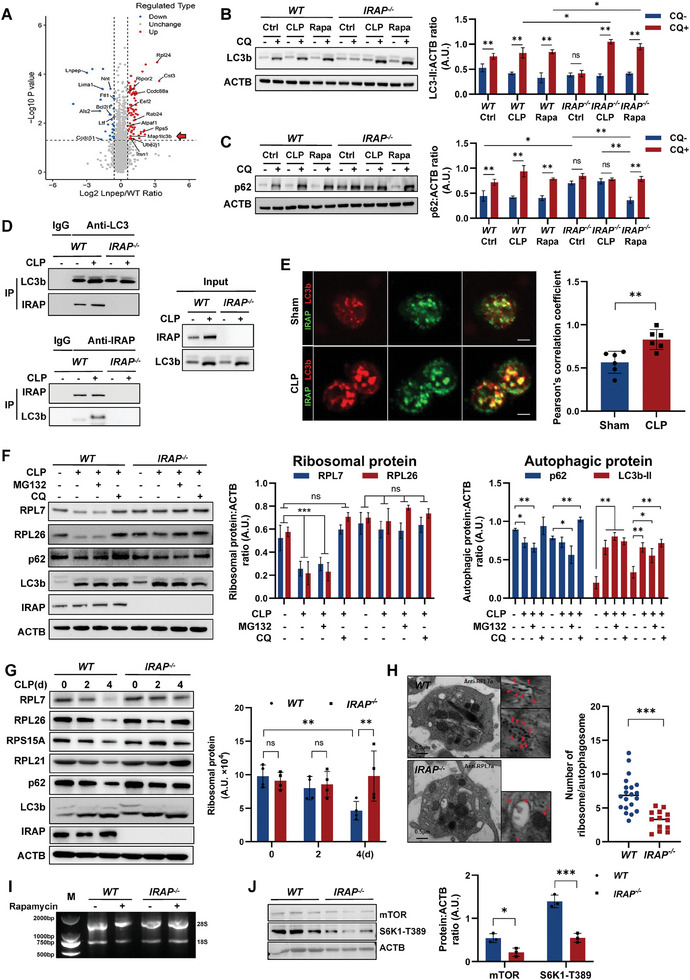
IRAP mediates autophagic degradation of ribosomal proteins. A) Volcano map of the differentially expressed proteins based on quantitative proteomic analysis. The x‐axis indicates the fold change (logarithmic conversion based on 2), and the y‐axis indicates the P‐value (logarithmic conversion based on 10). B,C) Immunoblotting for LC3B lipidation (LC3‐II) and SQSTM1/p62 accumulation‐degradation in *WT* and *IRAP^−/−^* platelets under standard culture conditions (ctrl) and upon 2d CLP or 100 nm rapamycin treatment. Where indicated, platelets were treated for 4 h with 50 µm of the lysosome inhibitor chloroquine (CQ). Densitometric quantification of the LC3‐II:ACTB ratio and p62:ACTB ratio are shown in the right panel (n = 3). D) IRAP immunoprecipitation assay of the lysates of platelets isolated from sham or CLP‐operated *WT* and *IRAP^−/−^* mice. Protein levels were tested using anti‐LC3b and anti‐IRAP antibodies. Isotype‐matched IgG served as the negative control. E) Immunofluorescence images of the co‐localization of IRAP (green) and LC3b (red) in platelets isolated from sham or CLP‐operated *WT* mice. Scale bars, 1 µm. F) Immunoblotting for ribophagy in CLP‐*WT* and CLP‐*IRAP^−/−^* platelets under inhibition of the proteasome (MG132, 10 mg kg^−1^ body weight, i.p.) or autophagy (CQ, 60 mg kg^−1^ body weight, i.p.). Densitometric quantification of the RPL7: ACTB ratio, RPL26: ACTB ratio, p62: ACTB ratio, and LC3b‐II: ACTB ratio are shown in the right panel (n = 3). G) Immunoblotting for total levels of indicated proteins in lysates of platelets isolated from *WT* and *IRAP^−/−^* mice for the indicated time points after CLP surgery. The bar graphs show the quantification of the indicated ribosomal proteins (n = 4). H) Representative immunoelectron microscopy (IEM) image for platelets isolated from CLP‐operated *WT* and *IRAP^−/−^* mice. The red arrows indicate RPL7a antibody‐immunogold labeling ribosomes inside an autophagosome. Quantification of the numbers of gold beads in different autophagosomes (n = 15–20). Scale bar, 500 nm. I) Gel electrophoresis of total RNA extracted from equal numbers of *WT* and *IRAP^−/−^* platelets treated with 250 nm rapamycin for 10 h. D) Representative immunoblots showing mTOR and phosphorylated S6K1‐T389 protein levels in *WT* and *IRAP^−/−^* platelets. ACTB was used as the loading control for normalization. *P <.05, **P <.01, ***P <.001. ns, no significance; A.U., arbitrary units; WT, wild‐type; IRAP, insulin‐regulated aminopeptidase; CLP, cecal ligation and puncture.

To clarify the reason for the notable accumulation of ribosomes in *IRAP^−/−^* platelets, we investigated the alterations in platelet machinery caused by IRAP deletion. Notably, Map1lc3b, a widely used autophagy marker involved in the formation of autophagosomes,^[^ ^34^ ^]^ was enriched among the differential proteins (**Figure** **5**A). This prompted us to investigate the autophagic function of *IRAP^−/−^* platelets. The ratio of light chain 3 beta 2 (LC3B2) to ACTB is an important indicator of autophagy. Sequestosome 1 (SQSTM1/p62), a ubiquitin‐binding protein delivered to lysosomes for degradation, is a widely used marker to monitor autophagic flux. *IRAP^−/−^* platelets did not respond effectively to chloroquine stimulation in normal and CLP conditions, exhibiting an accumulation of LC3b and p62 similar to that observed in *WT* platelets, and exhibited only partial recovery when strongly stimulated with rapamycin (Figure 5B,C). Immunofluorescence staining also demonstrated high levels of co‐localization of IRAP and LC3b in platelets collected from CLP mice (Figures 5E and S3B, Supporting Information), and the interaction between IRAP and LC3b was further verified using immunoprecipitation (Figure 5D). These results indicate that *IRAP^−/−^* platelets exhibit attenuated autophagic flux and less autophagosome formation (Figure S4D, Supporting Information), attributed to the loss of IRAP interaction with LC3b.

Ribosome protein degradation primarily occurs through the proteasome or autophagy pathway.^[^ ^35^, ^36^ ^]^ We subsequently collected platelets from the sham or CLP mice two days after surgery and treated them with different combinations of chloroquine and MG‐132 (a proteasome inhibitor). The results confirmed that autophagy, but not proteasome, is primarily responsible for ribosomal degradation in activated platelets (Figures 5F and S4E, Supporting Information). We subsequently focused on ribophagy, a form of selective autophagy, for the turnover of ribosomal proteins in response to the physiological needs of the cell.^[^ ^35^ ^]^ By analyzing the major r‐proteins RPL7, RPL21, RPL26, and RPS15A, we observed that they were depleted in the platelets at the onset of sepsis. However, this effect was blocked under IRAP deficiency conditions (Figure 5G). Immunoelectron microscopy revealed a significant reduction in PRL7A‐label ribosomes in the autophagosomes of *IRAP^−/−^* platelets (Figure 5H). In addition, we observed IRAP aggregation of increased co‐localization of IRAP with PRL7A in septic platelets via immunofluorescence staining (Figure S4F, Supporting Information). IRAP deficiency prevented the depletion of ribosomal RNA (Figure 5I). The mTORC1/S6K1 pathway activation, which can mediate ribosomal protein autophagic flux and ribosomal turnover during nutrition stress,^[^ ^37^, ^38^ ^]^ was significantly reduced in CLP‐*IRAP^−/−^* platelets compared to that in the CLP‐*WT* control group (Figure 5J). These results suggest that accumulated ribosomes in CLP‐*IRAP^−/−^* platelets are not offset by increased expression, and that IRAP directly regulates the autophagic degradation of r‐proteins through its interaction with LC3b during sepsis.

### 2.6 IRAP‐Mediated Ribophagy Aims to Provide Amino Acids for Reprogramming of Energy Metabolism in Septic Platelets

**Figure 6:**
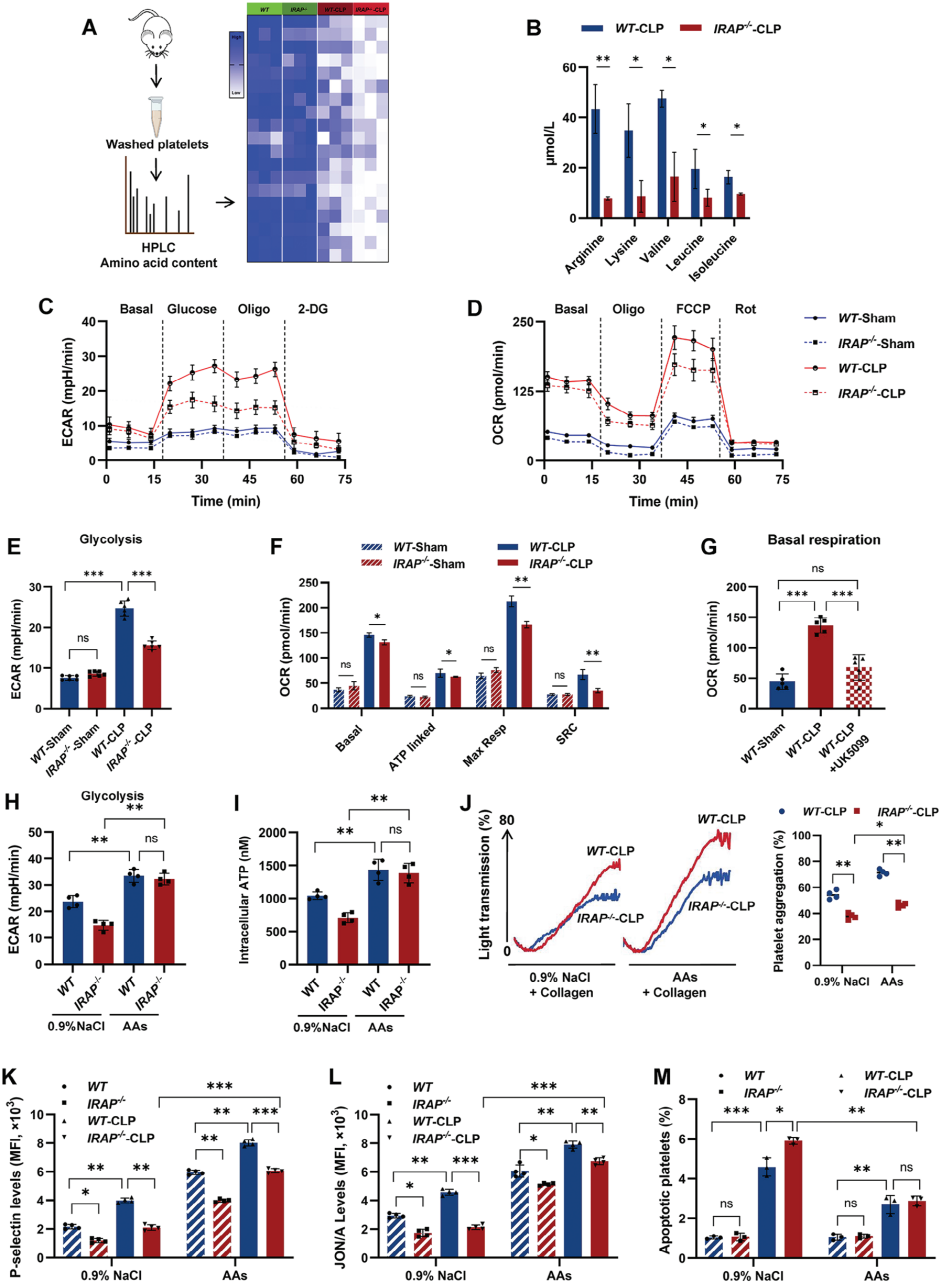
IRAP deficiency impedes energy metabolic reprogramming of septic platelets. A) Heatmap of the total amino acid levels in washed platelets collected from sham or CLP‐operated *WT* and *IRAP^−/−^* mice. Amino acid contents were determined by HPLC. B) Contents of basic amino acids (arginine and lysine) and branched‐chain amino acids (valine, leucine, and isoleucine) in platelets isolated from CLP‐operated *WT* and *IRAP^−/−^* mice(n = 3). C) ECAR in platelets isolated from sham and CLP‐operated *WT* and *IRAP^−/−^* mice (n = 3, 8 weeks old, male). D) OCR in platelets isolated from sham and CLP‐operated *WT* and *IRAP^−/−^* mice (n = 3, 8 weeks old, male). E) Maximum glycolysis calculated from the ECAR profile (n = 6). F) Basal respiration, ATP‐linked respiration, maximal respiration, and SRC calculated from the OCR profile (n = 3). G) Basal respiration calculated from the OCR profile (n = 5). H) Maximum glycolysis of *WT* and *IRAP^−/−^* platelets stimulated with 0.9% NaCl or 1 mm amino acids (AAs) mixture calculated from the ECAR profile (n = 4). 1 mm AAs mixture (0.3 mmol L^−1^ leucine, 0.1 mmol L^−1^ isoleucine, 0.1 mmol L^−1^ valine, 0.3 mmol L^−1^ arginine, and 0.2 mmol L^−1^ lysine). I) Quantification of intracellular ATP in lysates of *WT* or *IRAP^−/−^* platelets stimulated with 0.9% NaCl or 1 mm AAs mixture (n = 4). J) Aggregation of platelets from CLP‐operated *WT* and *IRAP^−/−^* mice with oral gavage treatment of 0.9% NaCl or 1 mg g^−1^ (body weight) of AAs mixture in response to 1.5 µg mL^−1^ collagen (n = 4). 10 g AAs mixture (weight ratio, 3:1:1:3:2 of leucine:isoleucine:valine:arginine:lysine) in 100 mL 0.9% NaCl. K,L) P‐selectin and JON/A exposure levels of platelets isolated from sham and CLP‐operated *WT* and *IRAP^−/−^* mice with oral gavage treatment of 0.9% NaCl or 1 mg g^−1^ (body weight) of AAs mixture in response to thrombin (n = 4). M) Percentage of apoptotic platelets isolated from sham and CLP‐operated *WT* and *IRAP^−/−^* mice with oral gavage treatment of 0.9% NaCl or 1 mg g^−1^ (body weight) of AAs mixture (n = 3). *P <.05, **P <.01, ***P <.001. ns, no significance; ECAR, extracellular acidification rate; OCR, oxygen consumption rate; SRC, spare respiratory capacity; IP, immunoprecipitation; WT, wild‐type; IRAP, insulin‐regulated aminopeptidase; CLP, cecal ligation and puncture.

To explore the effects of IRAP‐mediated ribophagy on platelet function, we focused on the products of ribosomal degradation. Ribosomes are abundant intracellular amino acid stores that can be recycled to maintain amino acids necessary for cell survival during stress.^[^ ^39^, ^40^ ^]^ Notably, HPLC analysis revealed that the amino acid content of CLP‐*IRAP^−/−^* platelets was generally lower than that of CLP‐*WT* platelets (**Figures** **6**A and S5A, Supporting Information). In particular, the levels of amino acids involved in energy metabolism,^[^ ^41^, ^42^, ^43^, ^44^ ^]^ such as basic amino acids (arginine and lysine) and branched‐chain amino acids (valine, leucine, and isoleucine), were significantly reduced in CLP‐*IRAP^−/−^* platelets (Figure 6B). There was no difference in the total levels of related amino acid transporters between CLP‐*WT* and CLP‐*IRAP^−/−^* platelets (Figure S5B, Supporting Information).

In line with the enrichment of mitochondrial function downregulation (Figure 4A) and the decrease in amino acids engaged in energy metabolism, we next measured and observed septic platelets exhibited higher extracellular acidification rate (ECAR) and oxygen consumption rate (OCR) values than those of naïve platelets, indicating that activated platelets exhibit increased glycolytic flux and mitochondrial activity (Figure 6C,D). Notably, mitochondrial activity, but not the glycolytic flux, promptly increased at the early stage of platelet activation, whereas glycolysis became the leading character during the continuous phase of platelet activation (Figure 6E,F). Consistent with a previous report indicating that glycolysis can maintain mitochondrial respiration in a pyruvate‐dependent manner during platelet activation,^[^ ^12^, ^45^ ^]^ we found that UK‐5099, an inhibitor of the mitochondrial pyruvate transporter,^[^ ^46^ ^]^ significantly reduced the respiratory parameters in septic platelets (Figures 6G and S5H, Supporting Information). IRAP deficiency notably inhibited glycolysis but not the initial mitochondrial activity (Figure 6C,D), reflecting the critical role of IRAP in glycolysis for prolonged platelet activation. In addition, we ruled out the effect of IRAP on platelet glucose uptake by measuring 2‐NBDG uptake and the localization of IRAP and GLUT3 in platelets (Figure S5C,E, Supporting Information). The expression levels of glycolysis‐related genes hypoxia‐inducible factor‐1a (*Hif1a*), hexokinase‐1 (*Hk1*), and hexokinase‐2 (*Hk2*) were compensatorily increased in CLP‐*IRAP^−/−^* platelets (Figure S5D, Supporting Information). Rescue experiments demonstrated that extensive supplementation with 1 mM amino acids mixture (0.3 mM leucine, 0.1 mm isoleucine, 0.1 mm valine, 0.3 mm arginine, and 0.2 mm lysine) markedly enhanced glycolysis and increased ATP production in *IRAP^−/−^* platelets stimulated with septic plasma (Figures 6H,I and S5F,G, Supporting Information). Targeted supplementation of branched‐chain amino acids and basic amino acids in vivo, also restored the activation and aggregation ability of *IRAP^−/‐^* platelets (Figure 6J–L). However, this was not a complete recovery owing to the role of IRAP in granule secretion. In vivo amino acid supplementation also effectively ameliorated *IRAP^−/−^* platelet apoptosis and prolonged their life span during sepsis (Figure 6M). Collectively, these data demonstrate that IRAP‐mediated ribophagy aims to regenerate activated platelets by providing specific amino acids for aerobic glycolysis and energy metabolic reprogramming.

### 2.7 S‐acylation is Crucial for IRAP to Exert Its Role in Facilitating Ribophagy and Platelet Hyperactivation

**Figure 7:**
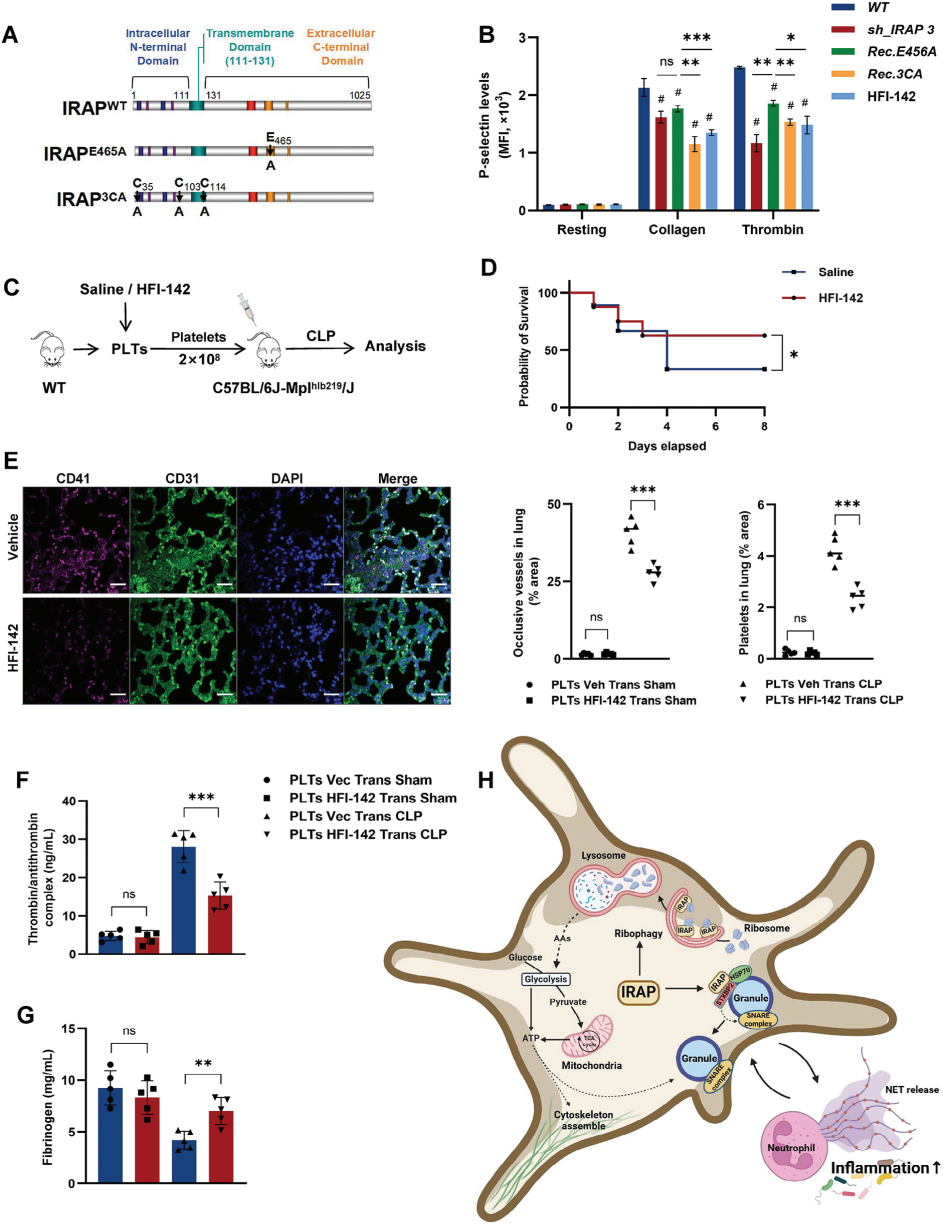
Blocking IRAP S‐acylation inhibits septic thrombosis and NETosis. A) Schematic drawing for the constructs of zyxin mutants lacking aminopeptidase activity (*IRAP E465A*), and protein S‐acylation sites C35A, C103A, and C114A (*IRAP 3CA*). B) Flow cytometry analysis of P‐selectin exposure levels in WT, IRAP KD, mutation (*IRAP E465A* and *IRAP 3CA*), and HFI‐142 treated platelets when resting or stimulated with thrombin (0.05 U/mL) or collagen (1 µg mL^−1^) (n = 3). C) Experimental design for the analysis of thrombosis after CLP surgery following the transfusion of either normal saline (control) or HFI‐142 treated platelets into *Mpl^−/−^* recipients. D) Survival curves of *Mpl^−/−^* mice transfused with platelets (treated with 0.9% NaCl or HFI‐142) after the CLP surgery (n = 10, 8 weeks, male). E) Immunofluorescence quantitative analysis of the ratio of occlusive vessels and platelets in the lungs of CLP‐operated platelet‐transfusion *Mpl^−/‐^* mice. Platelets, endothelial cells, and nuclei were stained with anti‐CD41 (purple), anti‐CD31 (green), and DAPI (blue). Scale bars, 50 µm. Quantification of CD41 staining area per field (n = 5). F,G) Plasma TAT and fibrinogen levels of *Mpl^−/‐^* mice transfused with sham and CLP‐operated platelets (n = 5). H) The schematic diagram illustrates that IRAP is involved in platelet granule secretion and ribosome autophagy, and it regulates platelet activation during sepsis. *P <.05; **P <.01; ***P <.001. ns, no significance; MFI, mean fluorescence intensity; DAPI, 4′,6‐diamidino‐2‐phenylindole; TAT, thrombin/antithrombin complex; WT, wild‐type; IRAP, insulin‐regulated aminopeptidase; CLP, cecal ligation and puncture.

As a type II transmembrane protein, the cytosolic domain of IRAP can undergo S‐acylation.^[^ ^47^, ^48^ ^]^ This process contributes to the hydrophobicity of IRAP, facilitating its participation in endosomal trafficking and its interaction with membrane components and specific proteins.^[^ ^49^, ^50^ ^]^ However, as IRAP is also a type of aminopeptidase,^[^ ^15^ ^]^ we further investigated whether its peptidase activity is directly responsible for platelet activation. To clarify the contributions of aminopeptidase activity and S‐acylation of IRAP to platelet activation, we reconstituted IRAP‐deficient CD34^+^ hematopoietic stem cells (HSCs) with two forms of IRAP variants: a full‐length protein lacking aminopeptidase activity due to a point mutation in the active site (*IRAP E465A*) and a full‐length protein with three cysteine residues (C35, C103, and C114) mutated to alanine (*IRAP 3CA*) to hinder protein S‐acylation (**Figures** **7**A and S6A,B, Supporting Information). Subsequently, following the induction of HSCs differentiation into megakaryocytes and subsequent platelet production, we collected and immunoblotted the platelets after 12 h of stimulation with rapamycin and/or LPS, as well as HFI‐142 (a pyridine compound that acts as an inhibitor of the catalytic domain of IRAP N‐terminal^[^ ^51^ ^]^). Similar to the HFI‐142 treatment group, platelets with *IRAP 3CA* displayed a marked reduction in ribophagy, whereas those with *IRAP E465A* exhibited the same level of ribophagy as the normal control (Figure S6C,D, Supporting Information). Correspondingly, the platelet activation index in the HFI‐142 treatment and *IRAP 3CA* groups also significantly decreased (Figures 7B and S6E–I, Supporting Information). These results demonstrate that S‐acylation is crucial for IRAP to exert its role in promoting platelet ribophagy, possibly because the high affinity of the S‐acylated domain of IRAP may facilitate endosomal enrichment of ribosomal proteins. Therefore, in activated platelets, IRAP is involved in endosomal trafficking by binding to autophagosomal‐membrane proteins, resulting in the lysosomal degradation of ribosomal proteins, with the aminopeptidase activity of IRAP playing a negligible role in this process.

To further evaluate the therapeutic effect of the IRAP on septic thrombosis, we performed a subsequent in vivo experiment to transfuse platelets pretreated with HFI‐142 or saline into *Mpl^−/−^* mice, followed by CLP surgery (Figure 7C). As expected, mice transplanted with HFI‐142 treated platelets exhibited few DIC indicators during the early stages of sepsis (Figures 7F,G and S7A–E, Supporting Information). Although the number of platelets began to decrease after two days, a higher survival rate was observed in the mice transfused with HFI‐142 treated platelets than in the control group mice (Figure 7D). Moreover, we detected reduced platelet accumulation in the lungs of mice transfused with HFI‐142 treated platelets (Figures 7E and S7F, Supporting Information). The NETosis induced by the co‐culture of HFI‐142 treated platelets and neutrophils was also significantly reduced (Figure S7G–I, Supporting Information). Hence, the inhibition of IRAP may be an effective strategy for the treatment of septic thrombosis.

## 3 Discussion

Platelets, the pivotal hemocytes in hemostasis and thrombosis, also serve as the first line of defense in the innate immune system against pathogenic invasions.^[^ ^6^, ^52^, ^53^ ^]^ Immune inflammatory thrombosis triggered by pathogen infection is a significant contributor to multi‐organ failure and mortality. IRAP, recognized as a crucial modulator of recycling endosomes, plays a critical role in orchestrating the immune cell response to pathogen infection. Modification of IRAP and its influence on endosomal dynamics may be intricately linked to the heightened risk of coagulation disorders and adverse clinical outcomes in infected patients. While the expression of IRAP in platelets has been identified, the extent to which IRAP modulates platelet function and septic thrombosis during pathogenic assaults remains an enigma.

Although platelets possess both the mitochondrial TCA cycle and oxidative phosphorylation (OXPHOS), aerobic glycolysis is the predominant form of energy metabolism for platelet activation.^[^ ^54^, ^55^ ^]^ Aerobic glycolysis is not only crucial for sustaining persistent platelet activation but also for maintaining mitochondrial metabolism during thrombosis.^[^ ^14^, ^41^, ^54^, ^56^ ^]^ Amino acid metabolism can assist in energy metabolism through various pathways. The oxidative degradation (deamination) of amino acids can produce NADH, which can be used in the electron transport chain to generate ATP, thereby affecting the ATP/ADP ratio.^[^ ^57^ ^]^ Some glucogenic amino acids, such as arginine, isoleucine, and valine, can be metabolized into pyruvate, α‐ketoglutarate, succinate, or oxaloacetate and then converted into glucose and glycogen through these carboxylic acids.^[^ ^58^, ^59^ ^]^ The metabolism of amino acids affects the intracellular ratios of NAD+/NADH and ATP/ADP, and changes in these ratios can alter the rates of glycolysis and the TCA cycle. Unlike glucose, which is accessible and abundant in platelets, these amino acids are insufficient and in high demand upon platelet activation.^[^ ^60^, ^61^ ^]^ Ribosomal proteins contain high levels of specific amino acids.^[^ ^35^, ^62^ ^]^

Ribosomes are the primary sites for protein translation, responsible for synthesizing nearly half of the cellular proteins.^[^ ^63^ ^]^ The process of protein translation and biogenesis in ribosomes requires high energy consumption, which is under strict surveillance.^[^ ^64^ ^]^ Therefore, the simultaneous downregulation of ribosome degradation and protein synthesis appears to be essential for cell survival, particularly under stress or nutrient starvation.^[^ ^35^ ^]^ The quality control process of ribosomes is attributed to selective autophagy, where ribosomes are selectively engulfed into autophagosomes and then degraded by lysosomes.^[^ ^65^ ^]^ In the present study, we revealed that IRAP‐mediated ribophagy aims to generate abundant amino acids necessary for aerobic glycolysis and subsequent mitochondrial metabolism. These findings can explain why platelets have an abundant energy supply for lifespan extension and prolonged activation during septic thrombosis. This study also unravels the enigma of why anucleate platelets, despite lacking transcription capacity, still retain abundant ribosomes that are theoretically regarded as “discarded”. In addition, we demonstrated that IRAP can also promote platelet granule secretion by facilitating SNARE complex assembly with the assistance of HSP70, further reflecting the important role of IRAP in platelet activation. Consequently, targeted inhibition of IRAP may significantly alleviate platelet hyperactivation. (Figure 7H)

We discovered that platelets possess a pathway for “making waste profitable” in which amino acids produced by ribosomal degradation are used to generate energy; these findings broaden our understanding of platelet organelle functions and energy metabolism. In septic thrombosis as well as many thrombotic diseases (e.g., stroke, shock, and diabetes), platelets are in a long‐term pre‐activated state.^[^ ^66^, ^67^, ^68^ ^]^ The substantial energy consumption may trigger IRAP‐mediated ribophagy to aid in energy metabolism. Therefore, targeting IRAP and energy metabolism may represent an alternative to conventional anti‐platelet drugs targeting enzymes or receptors,^[^ ^12^ ^]^ which are typically rapid, potent, and irreversible.^[^ ^69^ ^]^ Targeting IRAP and energy metabolism could provide a safe and reversible strategy, while retaining essential platelet functions. This is crucial for preventing bleeding and for patients requiring long‐term antithrombotic therapy.

## 4 Experimental Section

### Antibodies, Reagents, Mice, and More Methods

Detailed descriptions of antibodies, reagents, mice, and more methods are available in the supplemental methods.

### Sepsis Model: Cecal Ligation and Puncture (CLP) Assay

CLP operation was performed on 8‐week‐old male wild‐type (*WT*) C57BL/6J, *IRAP^−/−^*, and C57BL/6J‐Mpl^hlb219^/J mice, as previously described.^[^ ^23^ ^]^ Briefly, the mouse abdomen was disinfected with 75% medical alcohol, and CLP surgery was conducted under isoflurane anesthesia supplemented with oxygen. In the polymicrobial sepsis model, the cecum of mice was partially ligated and punctured using a 22‐gauge needle. Following puncture, an equal amount of feces was extruded, and the cecum was returned to its original position within the abdominal cavity. The sham group was subjected to the same surgical procedure without ligation and puncture. None of the experimental mice received antibiotics.

### Platelet Preparation, Aggregation, P‐Selectin Exposure, JON/A Binding, and Platelet Spreading

The preparation and stimulation of human and mouse platelets were performed as previously described.^[^ ^70^ ^]^ Briefly, for the platelet aggregation assay, 300 µL of platelets at a concentration of 3 × 10^8^/mL were used in response to collagen and thrombin. Platelets were then incubated with the PE‐conjugated JON/A (mouse), FITC‐conjugated PAC1 (human), FITC‐conjugated P‐selectin (mouse), or PE‐conjugated P‐selectin antibodies (human) in the presence of stimulants for 20 min at 25 °C. Subsequently, the levels of P‐selectin exposure, JON/A, or PAC1 binding were measured using flow cytometry. Platelet spreading on immobilized fibrinogen was performed as previously described.^[^ ^71^ ^]^ Platelets were stained using rhodamine‐conjugated phalloidin and visualized with a microscope (ZEISS 800; Zeiss, Jena, Germany). Five images were randomly selected and analyzed using ImageJ (National Institutes of Health, Bethesda, MD, USA).

### Measurement of Extracellular Acidification Rate (ECAR) and Oxygen Consumption Rate (OCR)

The Seahorse XFe 96 Extracellular Flux Analyzer (Agilent Technologies, Santa Clara, CA, USA) was employed to measure ECAR (mpH/min) and mitochondrial OCR (pmol/min).^[^ ^72^ ^]^ Platelets were seeded at a density of 1 × 10^8^ per well for ECAR and 2 × 10^7^ per well for OCR. For the ECAR assay, the Seahorse XF Glycolysis Stress Test Kit (Agilent Technologies) was used, with additions of 10 mm glucose, 1 µm oligomycin, and 50 mm 2‐DG to the wells at the indicated time points. Similarly, for the OCR assay, the Seahorse XF Cell Mito Stress Test Kit (Agilent Technologies) was used, with additions of 2 µm oligomycin, 0.25 µm carbonyl cyanide 4‐(trifluoromethoxy) phenylhydrazone (FCCP), and 1 µm rotenone/antimycin A to the wells at the indicated time points. Data analysis was conducted using the Wave Desktop Software (Seahorse Bioscience, North Billerica, MA, USA).

### Quantitative Proteomics Analysis

Mouse platelets were isolated from abdominal aorta blood at a concentration of 3 × 10^8^ per test, with three independent replicates for quantitative proteomics analysis using data‐independent acquisition mass spectrometry. The mass spectrometry proteomics data have been deposited in the ProteomeXchange Consortium via the PRIDE partner repository, under the dataset identifier PXD047357.

### High‐Performance Liquid Chromatography (HPLC)

HPLC was performed as previously described.^[^ ^73^ ^]^ Briefly, the AccQ‐Fluor kit (AQC, borate‐acetate buffer, and diluent reagent) and amino acid standard were purchased from Waters (Milford, MA, USA). A total of 10 µL platelet lysate was transferred to Eppendorf tubes for derivatization. Subsequently, 70 µL of the borate buffer and 20 µL of the derivatizing agent were added, followed by vortexing for 20 s. After resting for 1 min at 26 °C to ensure complete derivatization, the entire volume of the derivatized sample was transferred to the vials and incubated in a water bath at 60 °C for 10 min. A 5 µL aliquot was then injected into the HPLC system for further analyses.

### Statistics Analyses

Statistical significance was analyzed using unpaired two‐tailed *t*‐test and one‐way or two‐way analysis of variance tests. Values are presented as mean ± SD. Statistical significance was set at P < 0.05 (*P < 0.05, **P < 0.01, and ***P < 0.001). Statistical analyses were performed using GraphPad Prism 9.0.0 (Graph Pad Software, San Diego, CA, USA).

## Conflict of Interest

The authors declare no conflict of interest.

## Author Contributions

B.X. and X.Y. contributed equally to this work. J.W., S.C. J.Z., and B.X. designed the experiments, analyzed data, and wrote the paper. B.X. and X.Y. performed the experiments. X.Z., K.S., L.C., Z.F., Z.W., J.C., M.C., M.S., S.W., Y.X., and Q.L. helped with the experiments.

## Supporting information



Supporting Information

## Data Availability

The data that support the findings of this study are available from the corresponding author upon reasonable request.
