# Supplementary material for: IRAP Drives Ribosomal Degradation to Refuel Energy for Platelet Activation during Septic Thrombosis
Source: Adv Sci (Weinh). 2025 Jan 24;12(13):2411914. doi: 10.1002/advs.202411914 (PMC11967848; doi:10.1002/advs.202411914)
Supplement: Supplementary file 1 — Supporting Information [file ADVS-12-2411914-s001.pdf]

ADVANCED SCIENCE
Open Access

Supporting Information

for *Adv. Sci.*, DOI 10.1002/advs.202411914

IRAP Drives Ribosomal Degradation to Refuel Energy for Platelet Activation during Septic Thrombosis

Baichuan Xu, Xianpeng Ye, Kangfu Sun, Liang Chen, Zhaoyang Wen, Qigang Lan, Jun Chen, Mo Chen, Mingqiang Shen, Song Wang, Yang Xu, Xi Zhang, Jinghong Zhao, Junping Wang* and Shilei Chen**

Supporting Information for

IRAP drives degradation of ribosomes to refuel energy for platelet activation during septic thrombosis

Baichuan Xu, Xianpeng Ye, Kangfu Sun, Liang Chen, Zhaoyang Wen, Qigang Lan, Jun Chen, Mo Chen, Mingqiang Shen, Song Wang, Yang Xu, Xi Zhang, Jinghong Zhao, Junping Wang, Shilei Chen

Corresponding author:

Junping Wang, Email: wangjunping@tmmu.edu.cn

Shilei Chen, Email: chen.shilei@foxmail.com

Jinghong Zhao, Email: zhaojh@tmmu.edu.cn

The PDF file includes:

Materials and Methods

References: 1-11

Figure S1 to 7

Tables S1 to 3

Materials and Methods

KEY RESOURCES TABLE

Antibodies used in the study

REAGENT or RESOURCE	SOURCE	IDENTIFIER	DILUTION
**WB and CO-IP**			
Anti-IRAP antibody	Cell Signaling Technology	Cat# 3808S	1:1000
Anti-β-Actin antibody	Cell Signaling Technology	Cat# 4967S	1:1000
Anti-SQSTM1/p62 antibody	Cell Signaling Technology	Cat# 5114T	1:1000
Anti-HSP70 antibody	Abcam	Cat# ab181606	1:1000
Anti-STXBP2 antibody	Proteintech	Cat# 66238-1-lg	1:500
Anti-Syntaxin11 antibody	Abcam	Cat# ab216046	1:500
Anti-SNAP23	Abcam	Cat# ab3340	1:500
Anti-VAMP8/EDB antibody	Abcam	Cat# ab76021	1:500
Anti-RPS8 antibody	Abcam	Cat# ab201454	1:500
Anti-RPS9 antibody	Abcam	Cat# ab157125	1:500
Anti-RPS21 antibody	Abcam	Cat# ab254670	1:500
Anti-RPS15A antibody	Abcam	Cat# ab241420	1:500
Anti-RPL7 antibody	Abcam	Cat# ab72550	1:500
Anti-RPL15 antibody	Cell Signaling Technology	Cat# 29753S	1:500
Anti-RPL26 antibody	Abcam	Cat# ab59567	1:500
Anti-RPL21 antibody	Abcam	Cat# ab194664	1:500
Anti-RPL19 antibody	Abcam	Cat# ab224592	1:500
Anti-Phospho-p70 S6 Kinase 1-T389 antibody	ABclonal	Cat# AP0564	1:1000
Anti-mTOR antibody	Abcam	Cat# ab134903	1:1000
HRP Goat Anti-Rabbit IgG (H+L)	ABclonal	Cat# AS014	1:1000
HRP Goat Anti-Mouse IgG (H+L)	ABclonal	Cat# AS003	1:1000
Rabbit IgG	Abcam	Cat# ab172730	1:500
**Immunofluorescence**			
Anti-P-Selectin antibody	Santa cruz	Cat# sc-8419	1:100
Anti-VWF antibody	Santa cruz	Cat# 365712	1:100
Anti-PF4 antibody	Santa cruz	Cat# sc-73638	1:100
Anti-RPL7A antibody	Abcam	Cat# ab155147	1:100
Anti-LC3B antibody	Cell Signaling Technology	Cat# 43566	1:100

Anti-CD41	Abcam	Cat# ab134131	1:100
Anti-CD31	Abcam	Cat# ab222783	1:100
Anti-Histone H3 (citrulline R2 + R8 + R17)	Abcam	Cat# ab281584	1:100
Goat anti-mouse IgG (H+L)-Alexa Fluor (R) 488	Thermo Fisher	Cat# A-11029	1:700
Goat anti-rabbit IgG (H+L)-Alexa Fluor (R) 647	Thermo Fisher	Cat# A-21245	1:700
Goat anti-rabbit IgG (H+L)-Alexa Fluor (R) 594	Thermo Fisher	Cat# A-11037	1:700
**Flow cytometry**			
Anti-mouse CD107a-FITC	Proteintech	Cat# FITC-65050	1:100
Anti-mouse JON/A-PE	Emfret	Cat# M023-2	1:100
Anti-mouse CD62P (P-selectin) -PE	Biolegend	Cat# 148306	1:100
Anti-mouse CD41-APC	Biolegend	Cat# 133914	1:100
Anti-mouse CD42b-FITC	Emfret	Cat# M040-1	1:100
Anti-mouse ly6G-Alexa Fluor 488	Biolegend	Cat# 127626	1:100
Anti-mouse CD115-PE	Biolegend	Cat# 165003	1:100
Anti-human CD41-APC	Biolegend	Cat# 303709	1:100
Anti-human CD42b-FITC	Biolegend	Cat# 303903	1:100
PAC-1 antibody-FITC	Thermo Fisher	Cat# MA5-28564	1:100
Anti-human CD62P-PE	Biolegend	Cat# 986402	1:100
Streptavidin-APC	Biolegend	Cat# 405207	1:100
Anti-mouse CD107a-FITC	Proteintech	Cat# FITC-65050	1:100

Reagents and software used in the study

REAGENT or RESOURCE	SOURCE	IDENTIFIER
**Chemicals and recombinant proteins**		
DAPI	Thermo Fisher	Cat# 62248
Collagen	CHRONO-LOG	Cat# P/N385
Thrombin	CHRONO-LOG	Cat# P/N386
ADP	CHRONO-LOG	Cat# P/N384
Prostaglandin E1	MCE	Cat# HY-B0131
Fibrinogen	Biosharp	Cat# BS943
PI/RNase Staining Buffer	BD Pharmingen	Cat# 550825
Thiazole Orange	Sigma-Aldrich	Cat# 390062

Sulfo-NHS-LC-Biotin	Cayman	Cat# 13315
Lipopolysaccharide (LPS)	Sigma-Aldrich	Cat# L2880
HIF-142	Sigma-Aldrich	Cat# 5306130001
2-bromopalmitate	MCE	Cat# HY-111770
Rapamycin	MCE	Cat# HY-10219
Recombinant Human TPO	PEPROTECH	Cat# 300-18
Recombinant Murine TPO	PEPROTECH	Cat# 315-14
Recombinant Human SCF	PEPROTECH	Cat# 300-07
Recombinant Murine SCF	PEPROTECH	Cat# 250-03
Recombinant Human IL-3	PEPROTECH	Cat# 200-03
Recombinant Human IL-6	PEPROTECH	Cat# 200-06
BSA	Sigma-Aldrich	Cat# A1933
RIPA Buffer	Thermo Fisher	Cat# 89900
Protease Inhibitor Cocktail	Roche	Cat# 04693159001
Phosphatase Inhibitor Cocktail	Roche	Cat# 04906845001
SFEM medium	STEMCELL	Cat# 09650
HBSS	Gibco	Cat# 14025092
**Critical commercial assays**		
Mouse Platelet Factor4 (PF4) ELISA kit	Thermo Fisher	Cat# EEL120
ATP Assay Kit	Beyotime Biotechnology	Cat# S0026
Mouse fibrinogen ELISA kit	Abcam	Cat# ab213478
Mouse Thrombin-Antithrombin Complexes ELISA kit	Abcam	Cat# ab137994
Mouse TGF-beta ELISA	NOVUS	Cat# NBP1-92671
Mouse IL-1 beta Valukine ELISA	NOVUS	Cat# VAL601
Mouse Myeloperoxidase ELISA	abcam	Cat# ab275109
Seahorse XFp Extracellular Flux Cartridges	Agilent Technologies	Cat# C34917
XF Glycosis Stress test Kit	Agilent Technologies	Cat#103020-100
XF Mito Stress test Kit	Agilent Technologies	Cat#103015-100
EasySep Mouse CD117 (cKit) Positive Selection Kit	STEMCELL	Cat# 18757
EasySep ™ Human Cord Blood CD34 Positive Selection Kit II	STEMCELL	Cat# 17896
Neutrophil Isolation Kit	STEMCELL	Cat# 19762A
Dynabeads™ Co-Immunoprecipitation Kit	Thermo Fisher	Cat# 14321D

TB Green® Premix Ex Taq™ II (Tli RNaseH Plus)	Takara	Cat# RR820A
PrimeScript™ RT reagent Kit with gDNA Eraser (Perfect Real Time)	Takara	Cat# RR047A
RNAsimple Total RNA Kit	Tiangen	Cat# DP419
**Experimental models: Cell lines**		
HEK293T	Cell bank of the Chinese academy of sciences	N/A
MEG-01	Cell bank of the Chinese academy of sciences	N/A
**Oligonucleotides**		
5'-AGGTGTTTGAACTGTAGTTGACCT-3'		IRAP-KO-F1
5'-GGGAAAACGCTCTATCCTACAAGG-3'		IRAP-KO-R1
5'-ACTGAGCTGTATAAAGGCTTGAGG-3'		IRAP-KO-R2
5'-GCCAAGGTGAGGTGGATAGA-3'		Mpl-KO-F
5'-TCCTCTGATAGGGCCAAAGA-3'		Mpl-KO-R
5'-CGGAATGGGGAGCCTTTGG-3'		HK1-F
5'-GCCTTCCTTATCCGTTTCAATGG-3'		HK1-R
5'-TGATCGCCTGCTTATTCACGG-3'		HK2-F
5'-AACCGCCTAGAAATCTCCAGA-3'		HK2-R
5'-ACCTTCATCGGAAACTCC-3'		HIF1a-F
5'-CTGTTAGGCTGGGAAAAG-3'		HIF1a-R
5'-CATCTTCTTGTGCAGTGCCA-3'		GAPDH-F
5'-CGGCCAAATCCGTTCAC-3'		GAPDH-R
5'-TCTCGAGAATTCTCACGCGTGCCACCATGGAGTCCTTTACCA-3'		IRAP-E456A-F
5'-GATGATGGTGCCCTGGGCTAAGCGTAATCTGGAACATCGTATGGGTAGGCGTAGTCGGGCACGTC-3'		IRAP-E456A-R
5'-TCTCGAGAATTCTCACGCGTGCCACCATGGAGTCCTTTACCAATGATCGGCTT-3'		IRAP-3CA-F
5'-GATGATGATGGTGCCCTGGGCTATCCCGACAGCCACTGG-3'		IRAP-3CA-R
**Software and algorithms**		
Graphpad Prism 9.0	Graphpad software	N/A
MIST	Harvard Medical School	https://fgrtools.hms.harvard.edu/MIST

Image J	NIH	N/A
FlowJo v10.8.1	Three Star	N/A
ZEN 3.8	ZEISS	N/A
RStudio	N/A	www.rstudio.com

Mice

Wild-type (*WT*) C57BL/6J mice were purchased from the Beijing HFK Bioscience Co., Ltd. (Beijing, China). Mice deficient in IRAP (*IRAP*$^{-/-}$) were generated by Cyagen Biosciences, Inc (Guangzhou, China), littermate background mice (*IRAP*$^{+/+}$) were used as *WT* controls. C57BL/6J-Mpl^{hlb219}/J (*Mpl*$^{-/-}$) mice were obtained from the Jackson Laboratory (Bar Harbor, ME, USA). All mice were on a C57BL/6J background. Animals used in these experiments were male and female mice aged 8 to 14 weeks. All animal experiments were carried out in strict accordance with the recommendations in the Guide for the Care and Use of Laboratory Animals by the National Institutes of Health and were approved by the Animal Care and Use Committee of the Army Medical University (Chongqing, China; approval no. SYXC-2022-0018; project no. AMUWEC20230409).

Patient Recruitment and Isolation of Human Platelets

Critically ill patients with sepsis (n = 10) were recruited from the second affiliated hospital of Army Medical University between September 2021 and October 2024. All patients underwent clinically directed investigations to identify the pathogen causing infection, including bacterial, respiratory, and urinary cultures, and antigen testing, as directed by the treating clinical team. Healthy age- and sex-matched donors (n = 10) were also enrolled. Healthy donors had no known bleeding disorder, liver or kidney disease, cancer, or history of surgery or thrombotic event in the past 3 months and were not on antiplatelet or anticoagulant therapy. (Table 1) All donors signed informed consent forms, and the study was approved by the Ethics Committee of Army Medical University. This study was approved by the ethics committee of Xinqiao Hospital of Army Medical University (Institutional Review Board at No. 2018-006-01).

Venous blood samples were drawn from patients with sepsis on the first day of enrollment. Venous blood samples (3ml) were drawn from HS and patients with severe sepsis using blood collection tubes containing 3.8% trisodium citrate (w/v). Briefly, platelet-rich plasma (PRP) was centrifuged at 250×g at 25 °C for 15min. PRP was then treated with 100nM prostaglandin E1

(MCE, Cat# HY-B0131) and centrifuged at 1,000g for 5min. After discarding the supernatant, the platelet pellet was washed and resuspended in 3ml Hank's Balanced Salt Solution (HBSS; Gibco, Cat# 14025092). The purity of the platelet preparation was determined by FACS (ID7000; Sony Biotechnology Inc., Japan) analysis using platelet markers (>90% anti-human CD41, 1:100; Biolegend, Cat# 303709).

Quantitative real-time PCR analysis

Mouse platelets and megakaryocytes cDNA were used for quantitative real-time PCR analysis. Forty amplification cycles were performed with each cycle consisting of 95 °C for 5 seconds followed by 60 °C for 30 seconds. Amplification and dissociation curves generated by LightCycler® 96 software were used for gene expression analysis. Relative quantification of the expression of each gene was normalized to the gene expression of GAPDH.

Platelet Production From CBMCs-derived MKs

HumanCD34^{+}-derived megakaryocytes were cultured as previously described[1]. Cord blood mononuclear cells (CBMCs) were isolated by density gradient centrifugation with Lymphoprep (Stem Cell Techologies, Canada). CD34^{+} cells were isolated from CBMCs by EasySep™ Human Cord Blood CD34 Positive Selection Kit II (Stem Cell Techologies, Canada). Then isolated cells were cultured in SFEM medium (Stem Cell Techologies, Canada) with 1 % penicillin and streptomycin, 25 ng/mL of stem cell factor and 20 ng/mL of thrombopoietin (TPO) for 6 days. Cells were cultured with 50 ng/mL of TPO only from days 6 to 14, inducing them to differentiate into megakaryocytes. The medium was refreshed every 3 days. On 14th day, cultured platelets were collected from cell culture supernatant by centrifuging at 500×g for 10 min.

Sepsis Model: Cecal Ligation and Puncture (CLP) Assay

CLP operation was performed on male 8-week-old WT C57BL/6J mice, *IRAP*$^{-/-}$ mice, and *Mpl*$^{-/-}$ mice as previously described[2]. Briefly, mouse abdomen was disinfected with 75% medical alcohol, and CLP surgery was conducted under isoflurane with oxygen. For the polymicrobial sepsis model, the cecum of mice were partially ligated and then punctured with a 22-gauge needle. After puncturing, an equal amount of feces was squeezed out, and the cecum was then

placed back into the abdominal cavity. The wound was stitched, and the mice were injected with 1 mL of sterile saline immediately after surgery. Mouse survival was observed and recorded for 7 days after surgery. The sham group was subjected to the same surgical procedure without ligation and puncture. None of the experimental mice received antibiotics.

Platelet Transfusion Assay

Platelet transfusion assay was performed as previously described[3]. Platelets were enriched from *WT* and *IRAP*$^{-/-}$ mice, and they were injected into 8-week-old *Mpl*$^{-/-}$ mice by the tail veins (2×10^8 platelets per mouse). After injection, CLP operation and analysis of thrombosis were performed.

Hemostasis Model: Mouse Tail Bleeding Assay

To evaluate hemostasis, the bleeding time assay was performed as previously described[4]. Briefly, mice were anesthetized, and a 3 mm section of the tail tip was amputated. The injured tail was immediately immersed in 0.9% NaCl maintained at 37°C, and the bleeding time was recorded.

Vein Thrombosis Model: Inferior Vena Cava (IVC) Stenosis Assay

To evaluate vein thrombosis, the inferior vena cava stenosis model was performed as previously described[5]. Mice were anesthetized with 1% Nembutal and the IVC was ligated over a 31-g blunt needle with a 7.0 polypropylene suture. Forty-eight hours later, the IVC and the associated thrombus, were harvested, photographed, weighed and measured for thrombus length.

Platelet Preparation, Aggregation, P-selectin Exposure, JON/A Binding, and Platelet Spreading

The preparation and stimulation of human and mouse platelets were performed as described previously[4]. For the platelet aggregation assay, 300μL of platelets at a concentration of 3×10^8/mL were used in response to collagen and thrombin. Platelets were incubated with the PE-conjugated JON/A antibody (mouse), FITC-conjugated PAC1 antibody (human), FITC-conjugated P-selectin antibody (mouse) or PE-conjugated P-selectin antibody (human) in the presence of stimulants for 20 minutes at 25 °C. The levels of P-selectin exposure, JON/A or PAC1 binding were measured using flow cytometry. Platelet spreading on immobilized fibrinogen was completed as described previously[5]. Platelets were stained using rhodamine-

conjugated phalloidin and visualized with a microscope (ZEISS 800). Five images were chosen at random and analyzed with ImageJ.

Measurement of Neutrophil-Platelet and Monocyte-Platelet Aggregation

Samples of mouse orbital blood were collected at different time points after sham and CLP surgery, and anticoagulated whole blood was then treated with red blood cell lysate buffer (0.15 M NH4Cl, 1 mM $NaHCO_3$, 0.1 mM EDTA, pH=7.25) at 25°C for 10 minutes. Then the samples were centrifuged at 400×g for 5 minutes to obtain the supernatant for flow cytometry staining. Anti-mouse CD41, anti-mouse Ly6G and anti-mouse CD115 antibodies were used to label platelets, neutrophils, and monocytes, respectively. All samples were kept at 4°C for 30 minutes and analyzed using flow cytometry (ID7000; Sony Biotechnology Inc., Japan).

Platelet Secretion Analysis

For PF4 and ATP assays, platelet samples were collected after aggregation. The samples containing 5 mM EDTA were centrifuged for 10 minutes at 2100 rpm at 4°C to acquire the supernatants. For the PF4 ELISA, supernatant samples were diluted 1:200 and evaluated using the ELISA Kit of PF4. For the ATP assay, samples (1:20 dilution) were evaluated using the ATP Assay Kit. Manipulation was performed according to the guidance of manufacturer. For the LAMP1 assay, platelets were incubated with the FITC-anti-mouse LAMP1 antibody in the presence of thrombin for 20 minutes at room temperature and the level of LAMP1 expression was analyzed using flow cytometry (ID7000; Sony Biotechnology Inc., Japan).

ELISA

Levels of plasma fibrinogen, TAT, TGF-beta were measured using ELISA as suggested by the manufacturer.

Platelet Lifespan Assay

Platelet lifespan assays were performed as previously described.[6] Briefly, mice were injected with Sulfo-NHS-LC-Biotin intravenously, and orbital blood was collected at different time points. Biotin positive platelets were identified with APC-streptavidin by flow cytometry.

Western Blot and Inmunoprecipitation (IP)

IRAP was immunoprecipitated with the rabbit anti-IRAP antibody kindly provided by Susanna Keller (Virginia University, USA) or with rabbit anti-IRAP (D7C5, Cell Signaling Technology) bound on Dynabeads™ Protein G (Invitrogen) following the manufacturer's instructions. For detection by immunoblot, the antibodies are listed in KEY RESOURCES TABLE. All primary antibodies were used at 1:1000 dilution. All developing antibodies were goat anti-species coupled with HRP used at 1:1000 dilution. Cells were lysed in RIPA Buffer (Invitrogen) supplemented with protease inhibitor complete (Roche) and phosphatase inhibitor cocktails (Roche). Lysate supernatants were resuspended in 1× Laemmli buffer and were separated by SDS-PAGE using Criterion 7-12% acrylamide gels (BioRad) in Tris-Glycine-SDS buffer. The proteins were transferred on PVDF membranes (BioRad) using a Trans-Blot® Turbo™ Transfer System from BioRad. Membranes were blocked for 1 hour in 4% non-fat milk and incubated overnight with each antibody, washed extensively and incubated for 5 min with Clarity™ Western ECL Substrate (BioRad). The chemiluminescence signal was acquired using a ChemiDoc™ Imaging System and the quantification was realised with the Image Lab software (BioRad).

Immunofluorescence and Confocal Microscopy

Mouse platelets and primary megakaryocytes were prepared as described previously.[3] For immunofluorescence staining, cells were cultured on cover slips coated with 50 mg/mL fibrinogen at 37℃ and then spreading platelets were fixed in 4% paraformaldehyde for 10 minutes at 25 ℃. Fixed platelets were permeabilized in 0.2% Triton X-100 and blocked in 3% bovine serum albumin (BSA). After blocking, platelets were stained with anti-IRAP, anti-LC3b, and anti-RPL7a antibodies, primary megakaryocytes were stained with anti-IRAP, anti-P-selectin, anti-PF4, and anti-vWF antibodies at 4℃ over night. Fluorescent secondary antibody was incubated at 25 ℃ for 1 hour. Immunofluorescence images were captured using a laser scanning confocal microscope (ZEISS, LSM 800), and the 60× or 100× lens oil lens was used to obtain images. The images were analyzed using ZEN imaging software (Carl Zeiss, GmbH). For quantitative analysis of the ratio of occlusive vessels and platelets in lungs/livers, the paraffin sections were incubated and photographed by the corresponding antibody as described above. For in vitro NETosis immunofluorescence staining, platelets and neutrophils were co-cultured

and fixed to the cell crawl. The anti-citrullinated histone H3 antibody and DAPI were used. The images were captured and analyzed as described above.

Transmission Electron Microscopy (TEM)

For TEM, 4% glutaraldehyde in phosphate buffer (0.1 M, pH 7.4) was used to fix mouse platelets for 2 hours. Then, 2% osmium tetroxide was used to further fix the platelets after removing phosphate buffer. Platelets were embedded in epon and 2% uranyl acetate and lead citrate were used to stain the slices. Images were acquired with a CM-120 transmission electron microscope (FEI, OR, USA).

Measurement of ECAR and OCR

The Seahorse XFe 96 Extracellular Flux Analyzer (Agilent Technologies) was employed to measure ECAR (mpH/min) and mitochondrial OCR (pmol/min).[7] Platelets were seeded 1×10^8 per well for ECAR and 2×10^7 per well for OCR. 10 mM glucose, 1 μM oligomycin and 50 mM 2-DG in Seahorse XF Glycolysis Stress Test Kit (Agilent) were injected to wells at the indicated time points for ECAR assay. In the same way, 2 μM oligomycin, 0.25 μM Carbonyl cyanide 4-(trifluoromethoxy) phenylhydrazone (FCCP) and 1 μM rotenone/antimycin A in Seahorse XF Cell Mito Stress Test Kit (Agilent) were injected to wells at the indicated time points for OCR assay. Data analysis was conducted using Wave Desktop Software (Seahorse Bioscience).

Quantitative Proteomics Analysis

Mice platelets from abdominal aorta blood were prepared in 3×10^8 cells per test and three independent repeat groups for quantitative proteomics analysis with data-independent acquisition (DIA) mass spectrometry. The platelet sample for DIA analysis was denatured by 2% SDS buffer containing 50 mM DTT for 20 min at room temperature and then boiled at 100 °C for 5 min. The protein sample was alkylated for 1 hour at room temperature in the dark by addition of a final concentration of 200 mM iodoacetamide (IAA). Then, add five times the volume of pre-cooled acetone, and precipitate the proteins overnight in the refrigerator at -20 °C. Finally, the protein precipitates were resolved and digested by sequencing grade modified trypsin (Promega) at a protein-to-enzyme of 50:1 at 37 °C overnight. Tryptic peptides were collected by centrifugation for 20 min at 14,000 rcf at 20 °C. The tryptic peptides were treated with 1%

trifluoroacetic acid (TFA), and were purified using the C18 Ziptips, eluted with 0.1% TFA in 50~70% acetonitrile. The eluted peptides were lyophilized using a SpeedVac (ThermoSavant), and resuspended in 1% formic acid and 5% acetonitrile. The iRT peptides (Biognosys, Schlieren, Switzerland) were spiked into the sample prior to analysis according to manufacturer instructions. For MS/MS acquisition, DIA method was set 80 isolation windows according to the FWTH (Full width at half maximum) within alternated -45 V and -65 V. The full scan was set at a resolution of 1,200,000 over m/z range of 350 to 1,500; followed by DIA scans with resolution 30,000; CE: 30% ± 5; AGC target: 1e6 and maximal injection time: 54 ms. The DIA raw files were analyzed in Spectronaut X (Biognosys, Schlieren, Switzerland). The default settings were used for targeted analysis of DIA data in Spectronaut. All results were filtered by a Q value cutoff of 0.01 (corresponds to an FDR of 1%). The mass spectrometry proteomics data have been deposited to the ProteomeXchange Consortium via the PRIDE partner repository with the dataset identifier PXD047357. Pathway enrichment of differential proteins was studied with reference to KEGG (Kyoto Encyclopedia of Genes and Genomes, www.genome.jp/kegg/).

High Performance Liquid Chromatography (HPLC)

HPLC was performed as previously described[8]. AccQ-Fluor kit (AQC, borate-acetate buffer and diluent reagent) and amino acid standard were purchased from Waters® (Milford, MA, USA). Acetonitrile, methanol and α-aminobutyric acid (AABA) were purchased from Sigma-Aldrich (Milwaukee, WI, USA) and commercially available 0.9% (m/v) saline. Then 10.0 µl platelet lysate was transferred to Eppendorfs to begin derivatization, and 70.0 µl of the borate buffer and 20.0 µl of the derivatizing agent were added, vortexing for 20 s. After resting for 1 min at room temperature for complete derivatization, the entire volume of the derivatized sample was transferred to the vials and placed in a water bath at 60 °C for 10 min. A 5.0 µl aliquot was injected into the HPLC for further analysis. The analyses of the collected samples were carried out using the technique HPLC, using an Alliance HT Waters e2795® model, with a Nova-pakTM C18 (3.9×150 mm, 4.0 µm) Waters® reverse-phase column coupled to a fluorescence detector (Waters 2475®). The separation was performed using a mobile phase pH 5.0 and a concentration gradient, as described in Table 1. Mobile phase A was an acetate–phosphate buffer (eluent A, Waters), and mobile phases B and C were acetonitrile and ultrapure water, respectively. The flow of the mobile phase was 1.0 ml/min, and the system worked under controlled temperature at

36°C. The wavelengths used for detection were 250 nm for excitation and 395 nm for emission. Data were recorded using Empower software, version 3, Waters®. For standardization of the method, a Waters® 17 l-amino acids standards was used, with a concentration of 2,500 μm for all compounds except cystine, for which 1,250 μm was used: aspartic acid, serine, glutamic acid, glycine, histidine, arginine, threonine, alanine, proline, cystine, tyrosine, valine, methionine, lysine, isoleucine, leucine and phenylalanine. Linearity was determined using the Waters® standard containing the 17 l-amino acids. A series of standards were prepared at eight levels of concentration, ranging from 100 to 2,500 μm and, for cystine, from 50 to 1,250 μm. The regression line was performed with eight points, all in triplicate. The coefficients of determination of the curves were evaluated. The repeatability study was performed to verify the dispersal of the results obtained by the method of analysis. The results were evaluated according to the standard deviation and coefficient of variation (CV). Ten blank replicates were performed to estimate the limit of detection (LD) and the limit of quantification (LQ). The lower concentration of the analytes was detected and quantified by the methodology described in this study. The mean values and the standard deviation were used for the calculation, according to the formula recommended by Inmetro (DOQ-CGCRE-008, 2011).

Plasmid Construction and Transfection

The Knockdown of human IRAP by a commercial pGIPZ shRNA vector (https: //horizondiscovery.com/en/gene-modulation/knockdown/shrna/products/gipz-lentiviral-shrna). For the overexpression of mutants, the human *IRAP* full length sequence, *IRAP* mutants (*IRAP-3CA, IRAP-E465A*) sequence, were PCR amplified from HEK293T cell complementary DNA and cloned into commercial pHBLV vectors respectively. Plasmids were transfected into HEK293T cells using Lipo2000 (Invitrogen, USA), and the cells were lysed in Western and IP Lysis Buffer at 48 hours post-transfection. Human $CD34^+$ cells were infected with lentiviral expressing *IRAP* mutants (*IRAP-3CA, IRAP-E465A*), and vehicle vector (control) was performed by spinoculation (800×g; 90 min, 4°C) with a multiplicity of infection (MOI) of 200 on day 5 of cultivation. The expression levels of WT IRAP and IRAP mutants were determined by Western blot. Plasmids were constructed from Genewiz (Suzhou, China).

NETosis Experiment In Vitro

Platelet-induced NETosis was performed as previously described[10]. Briefly, anticoagulated whole blood was obtained from healthy volunteers, and then platelets and neutrophils were then isolated as previously described[11]. Platelets, which were preincubated with HFI-419 for 1 hour at 37°C, were then mixed with neutrophils and 0.2 U/mL thrombin for 2 hours at 37°C and 5% CO_2. The adherent cells were fixed with 4% paraformaldehyde for immunofluorescence staining. NET quantification in the cell culture supernatant was performed by using MPO-DNA ELISA. Briefly, the myeloperoxidase polyclonal antibody was used to coat 96-well plates overnight at 4°C. Then, 20 mL of supernatant sample plus 80 mL of incubation buffer including anti-DNA-POD was incubated in myeloperoxidase polyclonal antibodies coated 96-well plates and shaken for 2 hours at 300 rpm and 25°C the next day. Finally, peroxidase substrate (ABTS) was added to the 96-well plates and incubated for 20 minutes in the dark at room temperature. The MPO-DNA level was determined based on the absorbance measured at 405 nm according to the manufacturer's protocol.

Autophagy Regulation and Amino Acid Supplementation

For Immunoblotting on ribophagy, mice were treated with proteasome inhibitor (MG132, 10 mg/kg body weight, i.p.) or autophagy (Chloroquine, 60 mg/kg body weight, i.p.).

For assessment of maximum glycolysis, platelets were collected and stimulated with 0.9% NaCl or 1 mM amino acids (AAs) mixture (0.3 mmol/L Leu, 0.1 mmol/L Ile, 0.1 mmol/L Val, 0.3 mmol/L Arg, and 0.2 mmol/L Lys) calculated from the ECAR profile. For platelets aggregation analysis in response to 1.5 μg/mL collagen, mice were treated with 0.9% NaCl or 1 mg/g (body weight) of AAs mixture via oral gavage. 10g AAs mixture (weight ratio, 3:1:1:3:2 of Leu:Ile:Val:Arg:Lys) was resolved in 100 mL 0.9% NaCl.

Quantification and Statistical Analysis

Statistical analyses were performed with GraphPad Prism 9.0. Data are presented as the mean ± standard deviation (SD). An unpaired *t* test was used for comparisons between two conditions. For multiple comparisons, one-way ANOVA or two-way ANOVA was performed. Survival results were analyzed using the log-rank test. Values of $p \leq 0.05$ were considered statistically significant.

References

1. Xu Y, Wang S, Shen M, Zhang Z, Chen S, Chen F, et al. hGH promotes megakaryocyte differentiation and exerts a complementary effect with c-Mpl ligands on thrombopoiesis. Blood 2014;123:2250–2260. doi:10.1182/blood-2013-09-525402.

2. Rittirsch D, Huber-Lang MS, Flierl MA, Ward PA. Immunodesign of experimental sepsis by cecal ligation and puncture. Nat Protoc 2009;4:31–36. doi:10.1038/nprot.2008.214.

3. Yang M, Jiang H, Ding C, Zhang L, Ding N, Li G, et al. STING activation in platelets aggravates septic thrombosis by enhancing platelet activation and granule secretion. Immunity 2023;56:1013-1026.e6. doi:10.1016/j.immuni.2023.02.015.

4. Shi J, Tong R, Zhou M, Gao Y, Zhao Y, Chen Y, et al. Circadian nuclear receptor Rev-erbα is expressed by platelets and potentiates platelet activation and thrombus formation. European Heart Journal 2022;43:2317–2334. doi:10.1093/eurheartj/ehac109.

5. Yang K, Du C, Wang X, Li F, Xu Y, Wang S, et al. Indoxyl sulfate induces platelet hyperactivity and contributes to chronic kidney disease–associated thrombosis in mice. Blood 2017;129:2667–2679. doi:10.1182/blood-2016-10-744060.

6. Lan Q, Du C, Xiong J, Wu Y, Liao W, Liu C, et al. Renal Klotho safeguards platelet lifespan in advanced chronic kidney disease through restraining Bcl-xL ubiquitination and degradation. J Thromb Haemost 2022;20:2972–2987. doi:10.1111/jth.15876.

7. Chen Q, Xin M, Wang L, Li L, Shen Y, Geng Y, et al. Inhibition of LDHA to induce eEF2 release enhances thrombocytopoiesis. Blood 2022;139:2958–2971. doi:10.1182/blood.2022015620.

8. Nolasco DM, Fortes ICP, Valadares ER. Quantitative analysis of amino acids by HPLC in dried blood and urine in the neonatal period: Establishment of reference values. Biomedical Chromatography 2020;34:e4931. doi:10.1002/bmc.4931.

9. Montenont E, Bhatlekar S, Jacob S, Kosaka Y, Manne BK, Lee O, et al. CRISPR-edited megakaryocytes for rapid screening of platelet gene functions. Blood Adv 2021;5:2362–2374. doi:10.1182/bloodadvances.2020004112.

10. Caudrillier A, Kessenbrock K, Gilliss BM, Nguyen JX, Marques MB, Monestier M, et al. Platelets induce neutrophil extracellular traps in transfusion-related acute lung injury. J Clin Invest 2012;122:2661–2671. doi:10.1172/JCI61303.

11. Xu Y, Jiang H, Li L, Chen F, Liu Y, Zhou M, et al. Branched-Chain Amino Acid Catabolism Promotes Thrombosis Risk by Enhancing Tropomodulin-3 Propionylation in Platelets. Circulation 2020;142:49–64. doi:10.1161/CIRCULATIONAHA.119.043581.

Table S1.
Clinical Characteristics of Healthy Donors and Septic Patients.

	Healthy Donors (n=10)	Septic Patients (n=10)
**Age (mean, ±SD)**	51.32 (± 6.23)	55.23 (± 7.19)
**Male**	5/10	5/10
**Han nationality**	10/10	10/10
**BMI (mean, ±SD)**	23.20 (± 4.33)	24.13 (± 3.81)
**Diabetes**	3/10	3/10
**Hypertension**	1/10	2/10
**SOFA score (mean, ±SD)***	-	6 (± 2)
**Mechanical Ventilation**	-	1/10
**Shock requiring Vasopressors**	-	1/10
**90-day Survival**	10/10	10/10
**Aspirin**	-	2/10
**Platelet Count (mean, ±SD)****	209.13 (± 21.11)	286.91 (± 61.08)
**White Blood Count (mean, ±SD)*****	7.22 (± 3.10)	18.02 (± 11.25)
**Pneumonia**	-	5/10
**Staphylococcus**	-	4/10
**Klebsiella pneumoniae**	-	1/10

*SOFA score: sepsis-related organ failure assessment score;
**Reference Rangence: 159-439 K/μL;
***Reference Range: 4.3-11.3 K/μL.

Table S2.
Systemic clinical score to assess the severity of sepsis in CLP-mediated sepsis.

Test item	Score labeling
**Appearance**	0- Coat is smooth<br>1- Patched of piloerection<br>2- Piloerection<br>3- Piloerection with intermittent hunched posture
**Motor Activity**	0- Normal activity<br>1- Reduced activity<br>2- Activity is impaired
**Response to stimulation**	0- Normal response to auditory stimulus or touch<br>1- No response to auditory stimulus, normal response to touch<br>2- No response to auditory stimulus, slow response to touch<br>3- No response to auditory stimulus, no response to touch
**Eyes**	0- Open<br>1- Eyes not fully open, possibly with secretion<br>2- Eyes half closed, possibly with secretion

Table S3.
Differential Protein Expression of Proteomic Analysis.

Protein accession	Gene name	Lnpep/WT Ratio	Lnpep/WT P value	Regulated Type
P12246	Apcs	0.05459785	0.001649333	Down
Q8C129	Lnpep	0.12672162	8.86728E-05	Down
P03987	--	0.176413907	6.19508E-05	Down
Q920R0	Als2	0.278265522	0.00318942	Down
P01636	--	0.291451148	0.001099493	Down
Q3URS9	Ccdc51	0.302364821	0.046482907	Down
Q9ERG0	Lima1	0.317083547	0.000393623	Down
P01642	Gm10881	0.331113926	0.000411816	Down
P01635	Igkv12-41	0.335807209	6.13013E-05	Down
Q00724	Rbp4	0.388944763	0.021837822	Down
Q9DCV4	Rmdn1	0.457911041	0.011383454	Down
Q8K2F8	Lsm14a	0.506144882	0.004503746	Down
P62500	Tsc22d1	0.513333642	0.032595759	Down
Q3UUG6	Tbc1d24	0.517387923	0.046213954	Down
Q61941	Nnt	0.53298604	0.000401372	Down
Q64373	Bcl2l1	0.536034272	0.004566902	Down
P01646	--	0.553780064	0.001891916	Down
P01633	Igkv6-17	0.559478263	0.00715751	Down
P06330	--	0.572717272	0.007229415	Down
Q9DBG9	Tax1bp3	0.600733363	0.024119992	Down
P01878	--	0.605299771	0.03602829	Down
Q9JLT4	Txnrd2	0.6059922	0.017848392	Down
Q91WP6	Serpina3n	0.608983571	0.01101908	Down
Q8CIF4	Btd	0.632376427	0.018411527	Down
Q8BK67	Rcc2	0.643414042	0.000780613	Down
P28658	Atxn10	0.646111341	0.035192811	Down
P51655	Gpc4	0.647070096	0.012664532	Down
Q8CFX1	H6pd	0.648121725	0.002588531	Down
Q3TZM9	Alg11	0.655711554	0.043000928	Down
P29391	Ftl1	0.661837174	0.001003074	Down
P08071	Ltf	0.663349883	0.005599075	Down
Q811I0	Atpaf1	1.500381098	0.012847959	Up
P58252	Eef2	1.534572889	0.002561097	Up
P14148	Rpl7	1.559169963	0.003019849	Up
P19253	Rpl13a	1.592313072	0.021013284	Up
P62908	Rps3	1.594233102	0.000227219	Up
P62482	Kcnab2	1.619183101	0.000531954	Up
Q8BL06	Usp54	1.632474647	0.00401256	Up
Q64471	Gstt1	1.640066351	0.000227639	Up
P24549	Aldh1a1	1.641053338	0.000537158	Up
P70335	Rock1	1.653743952	0.017553475	Up

P97351	Rps3a	1.684060923	0.003806546	Up
P12970	Rpl7a	1.702246732	0.00057342	Up
O35945	Aldh1a7	1.704376908	0.000427915	Up
Q9ERV1	Mkrn2	1.709489886	0.042988553	Up
P70268	Pkn1	1.739661731	0.010168263	Up
Q80U16	Ripor2	1.754016336	0.000484873	Up
P62264	Rps14	1.757247851	0.001798005	Up
Q9DBD0	Ica	1.776298994	0.014013732	Up
P08553	Nefm	1.783512705	0.000307572	Up
P27659	Rpl3	1.791089336	0.000350735	Up
P62270	Rps18	1.802236575	0.000379331	Up
Q9CQV6	Map1lc3b	1.808128761	0.033949909	Up
Q9JJZ4	Ube2j1	1.822123535	0.041879276	Up
Q99JY3	Gimap4	1.823721116	0.042002187	Up
P62717	Rpl18a	1.833125077	0.000436852	Up
Q9Z0R4	Itsn1	1.854911157	0.044672908	Up
Q8BK48	Ces2e	1.856112716	0.000117751	Up
P62754	Rps6	1.86558634	0.002514638	Up
P61514	Rpl37a	1.867358599	0.004760443	Up
P62849	Rps24	1.86765739	0.013406823	Up
P62702	Rps4x	1.877609561	0.0018907	Up
O09167	Rpl21	1.885364562	0.022133751	Up
P25444	Rps2	1.886769791	0.001811947	Up
P07309	Ttr	1.888944378	0.023919574	Up
P62960	Ybx1	1.893592067	0.00385514	Up
P35979	Rpl12	1.921120227	0.000699097	Up
Q5SNZ0	Ccdc88a	1.959174773	0.000656901	Up
Q61233	Lcp1	1.959257627	0.020173375	Up
P14131	Rps16	2.010041459	0.000405079	Up
Q8C079	Strip1	2.024217387	0.014953007	Up
Q9CR57	Rpl14	2.038133147	0.000608835	Up
Q9CZX8	Rps19	2.05521554	0.004558414	Up
Q6ZWN5	Rps9	2.069401371	0.002772256	Up
Q9D8E6	Rpl4	2.121093786	0.011727337	Up
P62855	Rps26	2.15558596	0.027971776	Up
P35980	Rpl18	2.159047615	0.000551931	Up
P62911	Rpl32	2.17100288	0.044994772	Up
P62301	Rps13	2.191867115	0.000410848	Up
Q9CPR4	Rpl17	2.201892266	0.003372499	Up
P35290	Rab24	2.207037626	0.007730444	Up
Q9CY58	Serbp1	2.211655502	0.017670208	Up
P62889	Rpl30	2.222064278	0.00304336	Up
P62751	Rpl23a	2.232863068	0.000389075	Up
P62267	Rps23	2.273888026	0.000969593	Up
P53026	Rpl10a	2.280234666	0.000626232	Up
P41105	Rpl28	2.281928702	0.005800751	Up

P62281	Rps11	2.295298742	0.005544572	Up
P67984	Rpl22	2.31864287	0.014838427	Up
P62918	Rpl8	2.330217035	0.0002931	Up
Q9D1R9	Rpl34	2.344319113	0.000715677	Up
Q6ZWV7	Rpl35	2.385241228	0.000196723	Up
Q9EPU0	Upf1	2.422702644	0.007240052	Up
P84099	Rpl19	2.447122449	0.001207619	Up
Q6ZWV3	Rpl10	2.478301278	0.004153184	Up
P63325	Rps10	2.48486959	0.00052854	Up
P47911	Rpl6	2.505031313	0.001800538	Up
P61255	Rpl26	2.506235141	0.002815107	Up
P14115	Rpl27a	2.54934504	0.000645127	Up
P62830	Rpl23	2.590373131	0.000597131	Up
P47962	Rpl5	2.764955162	0.0045378	Up
Q64475	H2bc3	2.946929785	0.031821229	Up
P84244	H3-3b	3.056102457	0.041281403	Up
P15864	H1-2	3.360403519	0.026826692	Up
P62242	Rps8	3.387747951	0.022892008	Up
P61358	Rpl27	3.722024952	7.8627E-05	Up
P97461	Rps5	3.935060535	0.022521962	Up
Q9CXW4	Rpl11	4.156765158	0.019287975	Up
P62806	H4c16	4.307078948	0.029284527	Up
P01843	--	4.522921291	4.03648E-05	Up
Q8BP67	Rpl24	9.039416047	3.29261E-05	Up
P21460	Cst3	10.28643516	0.000191509	Up

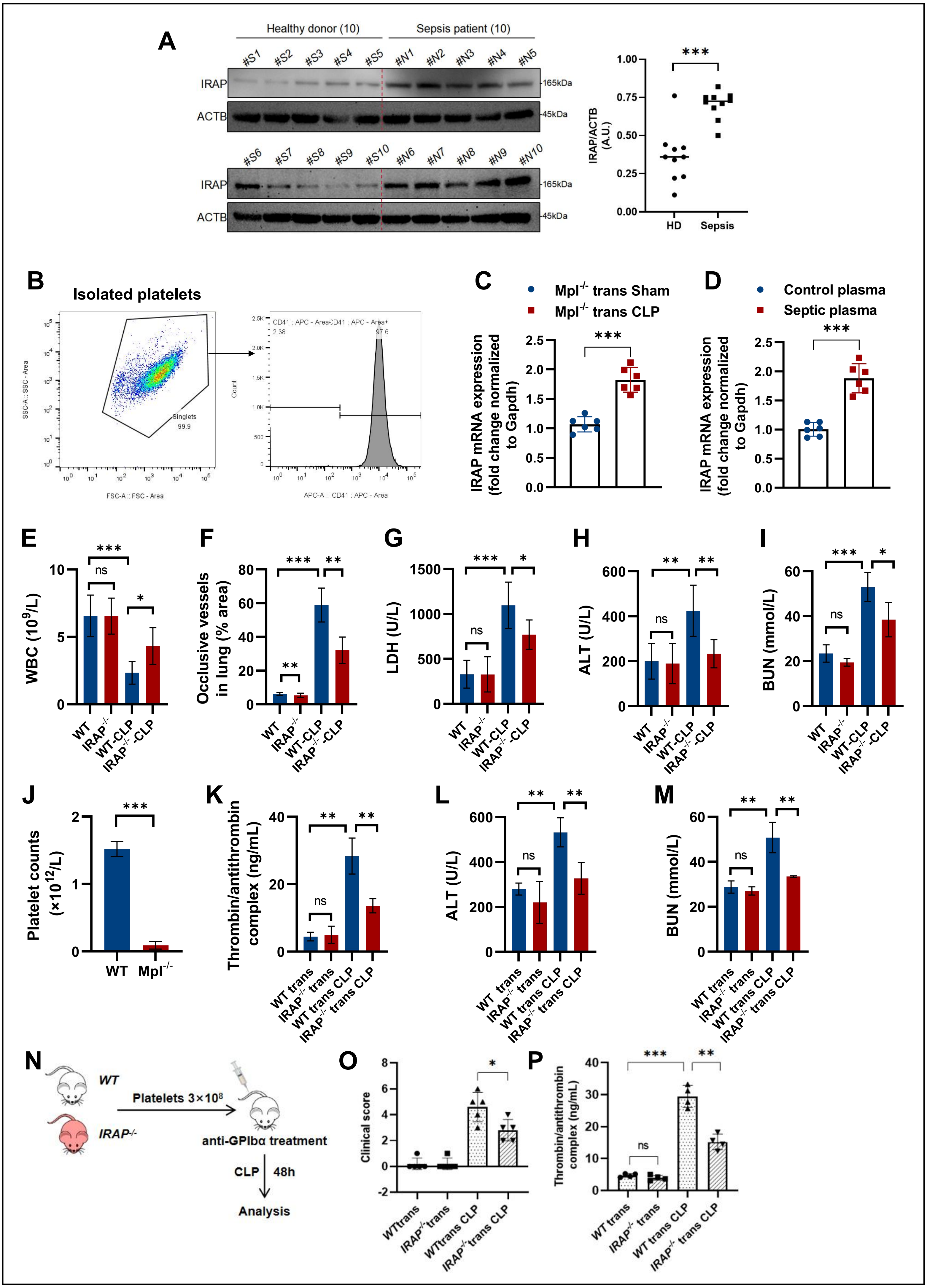
A
Healthy donor (10)
Sepsis patient (10)
IRAP
ACTB
165kDa
45kDa
IRAP/ACTB (A.U.)
HD
Sepsis
B
Isolated platelets
Singlets 99.9
C
Mpl-/- trans Sham
Mpl-/- trans CLP
IRAP mRNA expression (fold change normalized to Gapdh)
D
Control plasma
Septic plasma
E
WBC (10^9/L)
F
Occlusive vessels in lung (% area)
G
LDH (U/L)
H
ALT (U/L)
I
BUN (mmol/L)
J
Platelet counts (×10^12/L)
WT
Mpl-/-
K
Thrombin/antithrombin complex (ng/mL)
L
ALT (U/L)
M
BUN (mmol/L)
N
WT
IRAP-/-
Platelets 3×10^8
anti-GPIbα treatment
CLP 48h
Analysis
O
Clinical score
P
Thrombin/antithrombin complex (ng/mL)

Figure S1

Figure S1. Platelet IRAP deficiency results in milder signs of thrombosis and sepsis index.

(A) IRAP protein levels in platelets from healthy donors and septic patients.

(B) The purity of circulating platelets isolated from mice was detected by flow cytometry.

(C) IRAP mRNA expression was measured in platelets isolated from *Mpl*$^{-/-}$ mice transfused with WT or *IRAP*$^{-/-}$ platelets (n = 6).

(D) IRAP mRNA expression in human platelets incubated with normal or septic plasma (n = 6).

(E) WBC counts in sham and CLP-operated *WT* and *IRAP*$^{-/-}$ mice.

(F) The proportion of occlusive vessels in the lung of sham and CLP-operated *WT* and *IRAP*$^{-/-}$ mice.

(G-I) Blood serum biochemistry of lactate dehydrogenase (LDH) (G), liver enzyme alanine aminotransferase (ALT) (H), and blood urea nitrogen (BUN) (I) were measured in sham and CLP-operated *WT* and *IRAP*$^{-/-}$ mice (n = 5 in all groups) .

(J) Platelet counts in *WT* and *Mpl*$^{-/-}$ mice.

(K) Plasma thrombin/antithrombin complex (TAT) levels of sham and CLP-operated *WT* trans and *IRAP*$^{-/-}$ trans mice.

(L-M) Blood serum biochemistry of liver enzyme alanine aminotransferase (ALT) (L), and blood urea nitrogen (BUN)(M), were measured in *Mpl*$^{-/-}$ mice transfused with WT or *IRAP*$^{-/-}$ platelets.

(N) Schematic diagram of the transfer of platelets to platelet-depleted mice.

(O) Clinical score of sham and CLP-operated platelet-depleted mice transfused with WT or *IRAP*$^{-/-}$ platelets.

(P) Plasma thrombin/antithrombin complex (TAT) levels of sham and CLP-operated platelet-depleted mice transfused with WT or *IRAP*$^{-/-}$ platelets.

*p < .05; **p < .01; ***p < .001. ns, no significance.

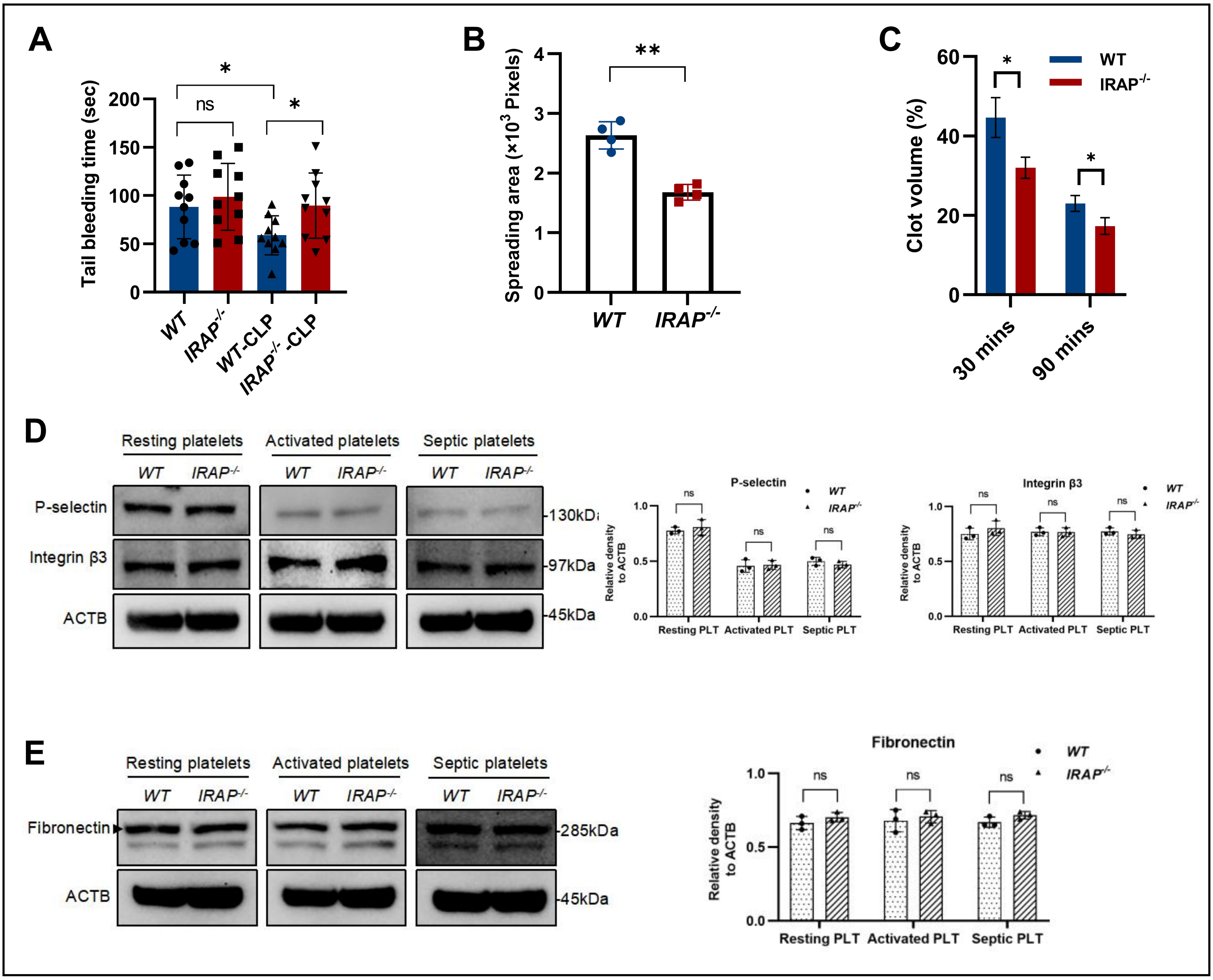
A
Tail bleeding time (sec)
200
150
100
50
0
ns
*
*
WT
IRAP-/-
WT-CLP
IRAP-/- -CLP
B
Spreading area (×10³ Pixels)
4
3
2
1
0
**
WT
IRAP-/-
C
Clot volume (%)
60
40
20
0
*
*
WT
IRAP-/-
30 mins
90 mins
D
Resting platelets
Activated platelets
Septic platelets
WT
IRAP-/-
P-selectin
Integrin β3
ACTB
130kDa
97kDa
45kDa
P-selectin
Integrin β3
Relative density to ACTB
ns
Resting PLT
Activated PLT
Septic PLT
E
Fibronectin
285kDa
45kDa
Relative density to ACTB
1.0
0.5
0.0

Figure S2

Figure S2. Role of IRAP in thrombosis and thrombopoiesis.

(A) Tail bleeding time in sham and CLP-operated *WT* and *IRAP*$^{-/-}$ mice (n = 10).

(B) Spreading of platelets isolated from sham and CLP-operated *WT* and *IRAP*$^{-/-}$ mice on immobilized fibrinogen. Areas (pixel numbers) of three random fields of *WT* and *IRAP*$^{-/-}$ platelets (n = 5).

(C) Clot retraction was triggered after addition of thrombin (1 U/mL). Data were quantified as clot volume (%) (n = 3).

(D) The levels of total P-selectin and beta 3 integrin in resting, activated, and septic *WT* and *IRAP*$^{-/-}$ platelets.

(E) The levels of total fibrinogen in resting, activated, and septic *WT* and *IRAP*$^{-/-}$ platelets.

*p < .05; ***p < .001. ns, no significance.

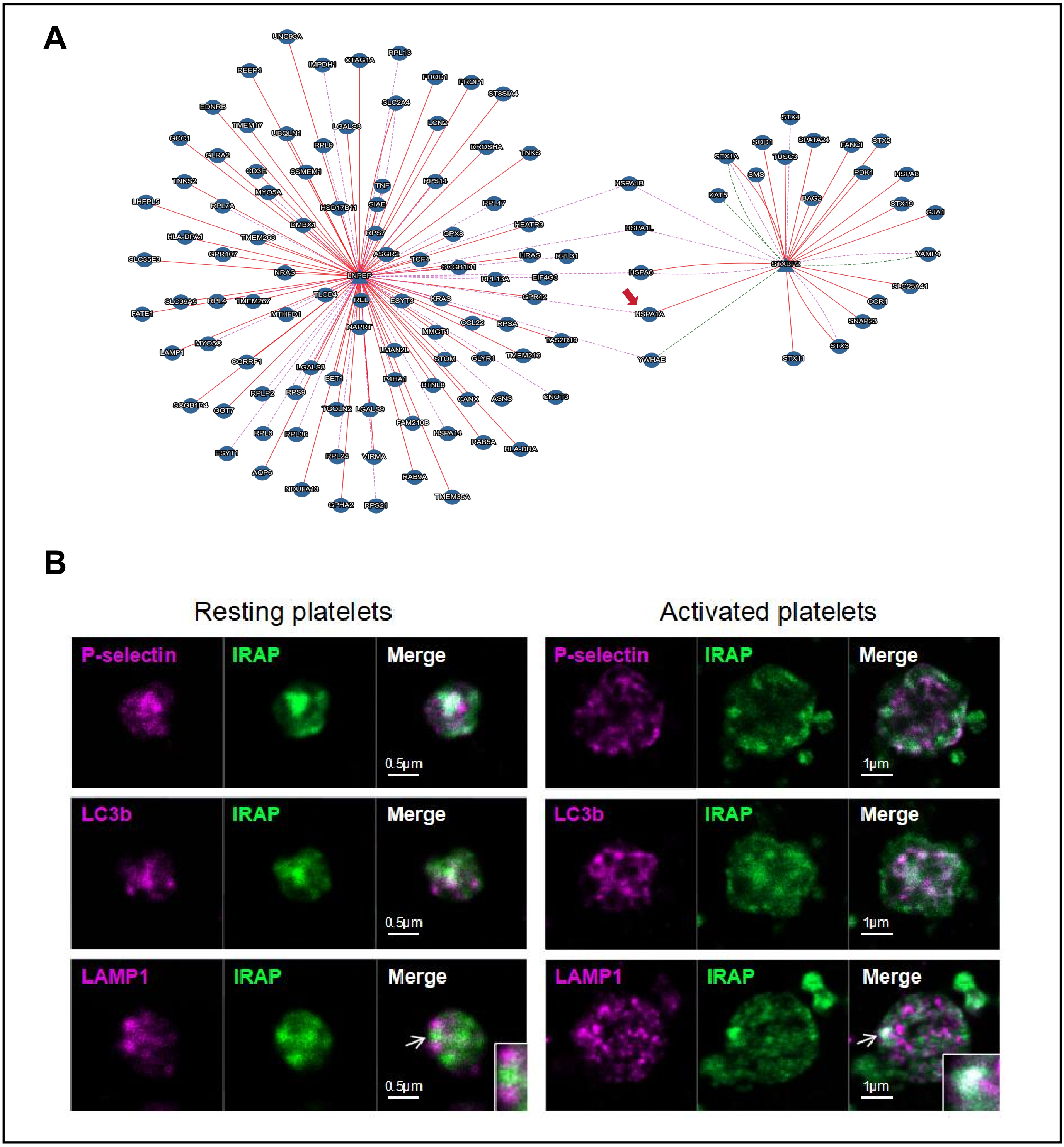

A
UNC93A
IMPDH1
CTAG1A
RPL13
REEP4
FHOD1
PROP1
ST8SIA4
SLC2A4
EDNRB
LCN2
TMEM17
UBQLN1
LGALS3
GCC1
RPL9
DROSHA
TNKS
GLRA2
CD3E
SSMEM1
TNKS2
MYO5A
TNF
RPS14
LHFPL5
RPL7A
HSD17B11
SIAE
RPL17
DMBX1
HEATR3
HLA-DPA1
TMEM263
RPS7
GPX8
GPR107
ASGR2
TCF4
HRAS
RPL31
SLC35E3
SCGB1D1
NRAS
LNPEP
RPL13A
EIF4G3
TLCD4
KRAS
GPR42
SLC39A9
RPL4
TMEM267
REL
ESYT3
FATE1
MTHFD1
CCL22
RPSA
NAPRT
MMGT1
TAS2R19
LAMP1
MYO5C
LMAN2L
CGRRF1
STOM
GLYR1
TMEM246
LGALS8
BET1
P4HA1
BTNL8
CNOT3
RPLP2
RPS9
CANX
ASNS
SCGB1D4
GGT7
TGOLN2
LGALS9
FAM210B
RPL6
RPL36
HSPA14
RAB5A
HLA-DRA
ESYT1
RPL24
VIRMA
AQP6
RAB9A
NDUFA13
TMEM35A
GPHA2
RPS21
HSPA1B
HSPA1L
HSPA6
HSPA1A
YWHAE
STX4
SOD1
SPATA24
FANCI
STX2
STX1A
TUSC3
SMS
PDK1
HSPA8
KAT5
BAG2
STX19
GJA1
STXBP2
VAMP4
SLC25A41
CCR1
SNAP23
STX3
STX11
B
Resting platelets
Activated platelets
P-selectin
IRAP
Merge
0.5µm
1µm
LC3b
LAMP1

Figure S3

Figure S3. IRAP is involved in platelet secretory function.

(A) IRAP interact with STXBP2, predicted by the Molecular Interaction Search Tool (MIST).

(B) IRAP co-localizes with alpha-granules, autophagosomes, and lysosomes in both resting and activated platelets.

*p < .05; **p < .01; ***p < .001. ns, no significance.

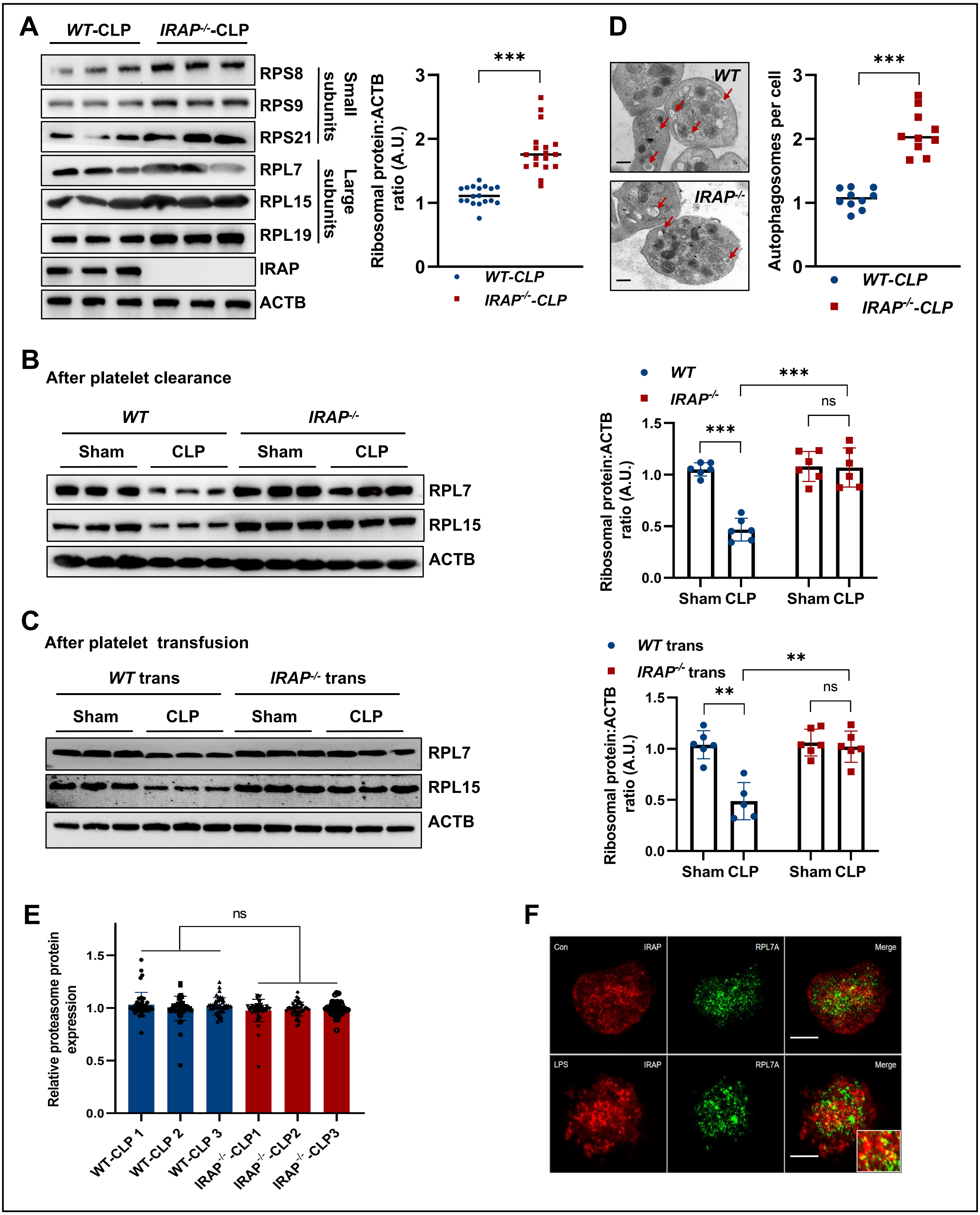
A
WT-CLP
IRAP-/--CLP
RPS8
RPS9
RPS21
Small subunits
RPL7
RPL15
RPL19
Large subunits
IRAP
ACTB
***
Ribosomal protein:ACTB ratio (A.U.)
WT-CLP
IRAP-/--CLP
D
WT
IRAP-/-
***
Autophagosomes per cell
WT-CLP
IRAP-/--CLP
B
After platelet clearance
WT
IRAP-/-
Sham
CLP
Sham
CLP
RPL7
RPL15
ACTB
WT
IRAP-/-
***
ns
***
Ribosomal protein:ACTB ratio (A.U.)
Sham CLP
Sham CLP
C
After platelet transfusion
WT trans
IRAP-/- trans
Sham
CLP
Sham
CLP
RPL7
RPL15
ACTB
WT trans
IRAP-/- trans
**
ns
**
Ribosomal protein:ACTB ratio (A.U.)
Sham CLP
Sham CLP
E
ns
Relative proteasome protein expression
WT-CLP 1
WT-CLP 2
WT-CLP 3
IRAP-/- -CLP1
IRAP-/- -CLP2
IRAP-/- -CLP3
F
Con
IRAP
RPL7A
Merge
LPS
IRAP
RPL7A
Merge

Figure S4

Figure S4. Platelet ribosomes degrade independently of the proteasome pathway.

(A) Protein levels of three proteins of the small ribosomal subunit and three proteins of the large ribosomal subunit in platelets from CLP-operated *WT* and *IRAP*$^{-/-}$ mice analyzed by western blot.

(B) Platelets were eliminated by tail IV injection of anti-CD42b antibody (2 mg/g weight) in *WT* and *IRAP*$^{-/-}$ mice. Platelets were collected on the 4th day after CLP surgery and the ribosomal protein content was measured. The bar graphs show the quantification of the indicated ribosomal proteins (n = 3).

(C) 2×10^8 *WT* and *IRAP*$^{-/-}$ platelets were transfused to *mpl*$^{-/-}$ mice. Platelets were collected on the 4th day after CLP surgery and the ribosomal protein content was measured. The bar graphs show the quantification of the indicated ribosomal proteins (n = 3).

(D) The quantitative analysis of autophagosomes being present in platelets from CLP-operated *WT* and *IRAP*$^{-/-}$ mice (n = 10).

(E) Total proteasome proteins in platelets of CLP-operated *WT* and *IRAP*$^{-/-}$ mice based on quantitative proteomic analysis.

(F) Immunofluorescence staining for IRAP (red) and RPL7A (green) in spreading platelets treated with/without LPS. Scale bar, 1 μm.

**p < .01; ***p < .001. ns, no significance.

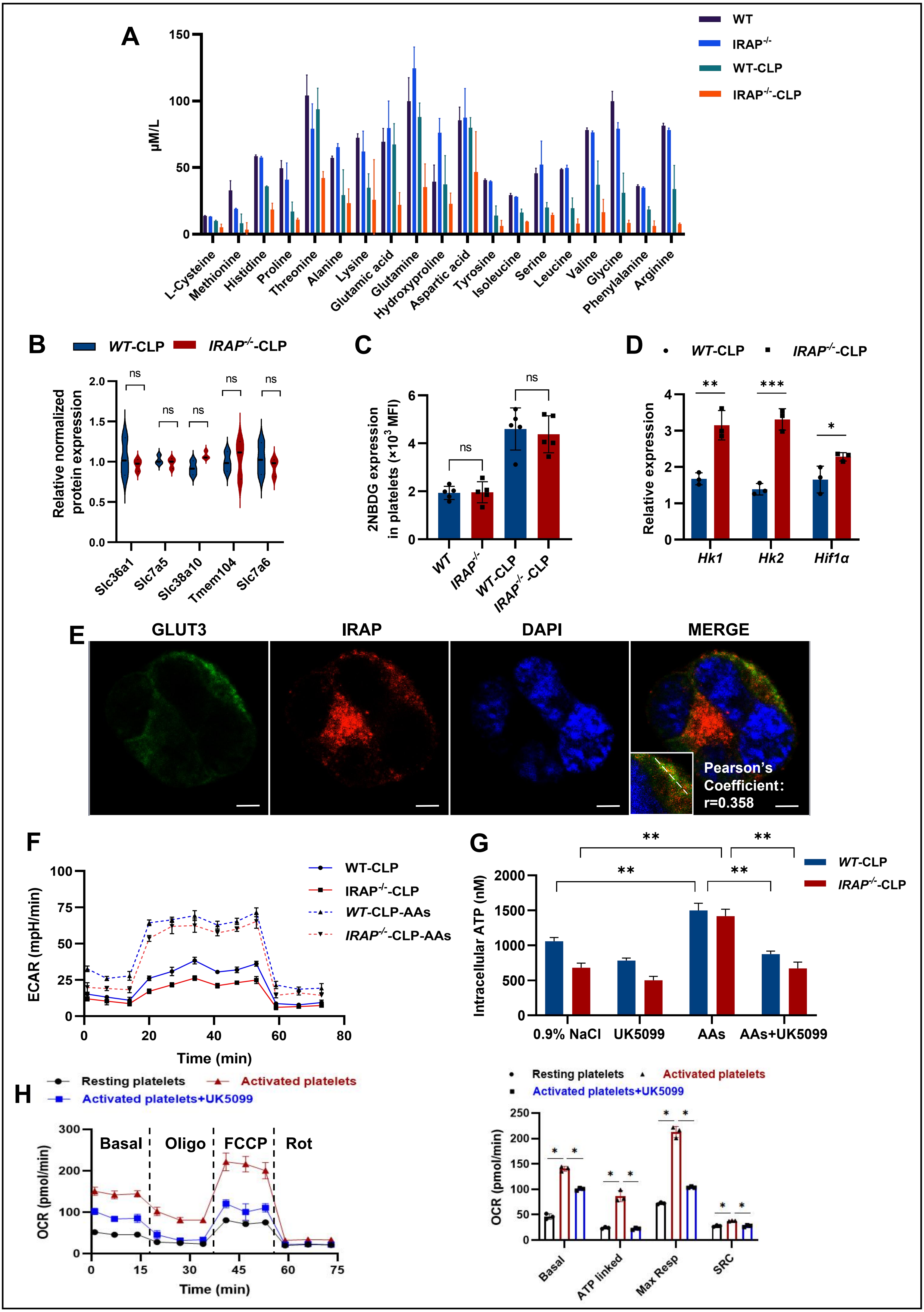
A
WT
IRAP-/-
WT-CLP
IRAP-/--CLP
μM/L
L-Cysteine
Methionine
Histidine
Proline
Threonine
Alanine
Lysine
Glutamic acid
Glutamine
Hydroxyproline
Aspartic acid
Tyrosine
Isoleucine
Serine
Leucine
Valine
Glycine
Phenylalanine
Arginine
B
WT-CLP
IRAP-/--CLP
Relative normalized protein expression
Slc36a1
Slc7a5
Slc38a10
Tmem104
Slc7a6
C
2NBDG expression in platelets (×10³ MFI)
WT
IRAP-/-
WT-CLP
IRAP-/- -CLP
D
WT-CLP
IRAP-/--CLP
Relative expression
Hk1
Hk2
Hif1α
E
GLUT3
IRAP
DAPI
MERGE
Pearson's Coefficient: r=0.358
F
WT-CLP
IRAP-/--CLP
WT-CLP-AAs
IRAP-/--CLP-AAs
ECAR (mpH/min)
Time (min)
G
WT-CLP
IRAP-/--CLP
Intracellular ATP (nM)
0.9% NaCl
UK5099
AAs
AAs+UK5099
H
Resting platelets
Activated platelets
Activated platelets+UK5099
Basal
Oligo
FCCP
Rot
OCR (pmol/min)
Time (min)
Basal
ATP linked
Max Resp
SRC

Figure S5

Figure S5. IRAP regulates platelet amino acid content without interfering with amino acid and glucose uptake.

(A) Amino acids levels in washed platelets collected from CLP or sham *WT* and *IRAP*$^{-/-}$ mice.

(B) Protein contents of amino acid transporters in platelets of CLP-*WT* or CLP-*IRAP*$^{-/-}$ mice.

(C) Mean fluorescence intensity of 2-NBDG uptake in platelets isolated from sham and CLP-operated WT and IRAP$^{-/-}$ mice (n = 5).

(D) qPCR assessment of mRNA expression of Hk1, Hk2, and Hif1a in platelets isolated from CLP-operated WT and IRAP$^{-/-}$ mice (n = 3).

(E) Immunofluorescence staining for IRAP (red) and GLUT3 (green). Scale bar, 20 μm.

(F) ECAR in platelets of *WT* or *IRAP*$^{-/-}$ mice treated with 0.9% NaCl or amino acid mixture (AAs: arginine, lysine, valine, leucine and isoleucine).

(G) Quantification of intracellular ATP in lysates of platelets of *WT* or *IRAP*$^{-/-}$ mice stimulated with LPS, and treated with/without AAs and UK5099.

(H) OCR in resting platelets, activated platelets, and activated platelets treated with UK5099.

*p < .05; **p < .01; ***p < .001. ns, no significance.

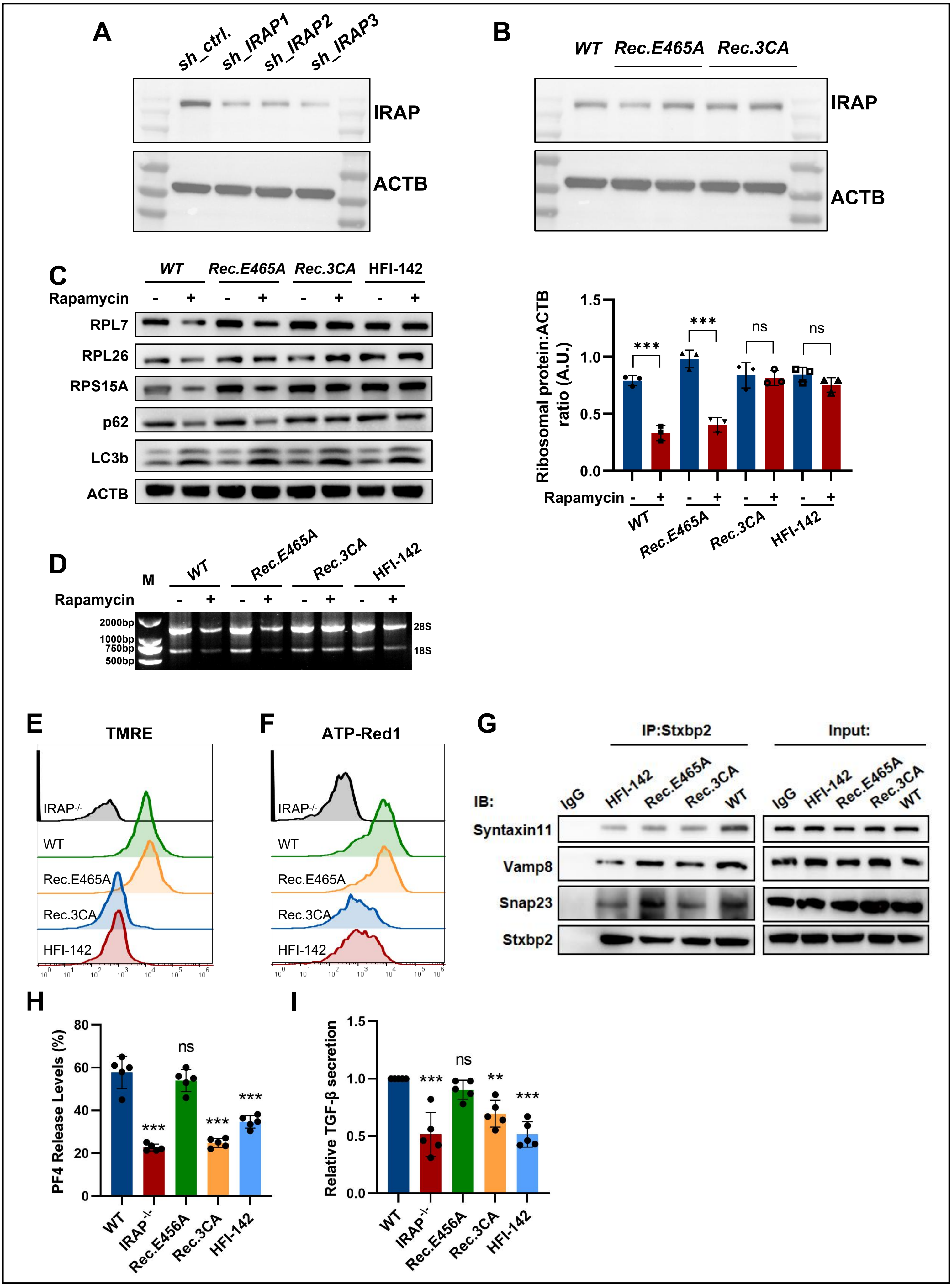
A
sh_ctrl.
sh_IRAP1
sh_IRAP2
sh_IRAP3
IRAP
ACTB
B
WT
Rec.E465A
Rec.3CA
IRAP
ACTB
C
WT
Rec.E465A
Rec.3CA
HFI-142
Rapamycin
RPL7
RPL26
RPS15A
p62
LC3b
ACTB
Ribosomal protein:ACTB ratio (A.U.)
***
***
ns
ns
D
M
Rapamycin
2000bp
1000bp
750bp
500bp
28S
18S
E
TMRE
IRAP-/-
WT
Rec.E465A
Rec.3CA
HFI-142
F
ATP-Red1
G
IP:Stxbp2
Input:
IB:
IgG
Syntaxin11
Vamp8
Snap23
Stxbp2
H
PF4 Release Levels (%)
WT
IRAP-/-
Rec.E456A
Rec.3CA
HFI-142
I
Relative TGF-β secretion

Figure S6

Figure S6. S-acylation modification is the basis of IRAP involved in the regulation of platelet activation.

(A) Assessment of knockdown efficiency in the human lymphoma cell line K562 by Western blot. Representative immunoblot of non-silencing (*sh_ctrl.*) and shRNAs targeting IRAP (*sh_IRAP 1-3*) in lysates of K562 cells (10 μg/sample) to test Knockdown efficiency. *sh_IRAP 3* was used for subsequent human MK experiments. ACTB serves as a loading control.

(B) Recombination expression efficiency of WT and IRAP mutants (*E465A, 3CA*) in MKs cultured from human cord blood $CD34^+$ cell. ACTB serves as a loading control.

(C) Loss of IRAP S-acylation inhibits the degradation of ribosomes caused by treatment with rapamycin. WT, mutation (IRAP E465A and IRAP 3CA), and HFI-142 treated platelets were stimulated with rapamycin at the indicated time points. Platelet lysates were analyzed by immunoblotting for the total levels of the indicated proteins.

(D) Mutation (IRAP 3CA) or HFI-142 treatment inhibits the degradation of 28S and 18S rRNA caused by rapamycin treatment. WT and IRAP mutant platelets were stimulated with 250 nM Rapamycin for 10 h and total RNA was extracted and analyzed via gel electrophoresis. RNA from equal numbers of platelets was loaded in each lane.

(E, F) Mitochondrial function (E), and ATP levels (F) in cultured *WT* and *IRAP*-mutant platelets.

(G) IP-Stxbp2 assay of the lysates from *WT* and *IRAP*-mutant platelets. The SNARE complex protein expression in platelets was tested using anti-Syntaxin11, anti-VAMP8, and anti-SNAP23 antibodies.

(H, I) Release levels of PF4 (H), and TGF-β (I) in WT, HFI-142 treated and IRAP-mutant platelets.

**$p < .01$, ***$p < .001$. ns, no significance.

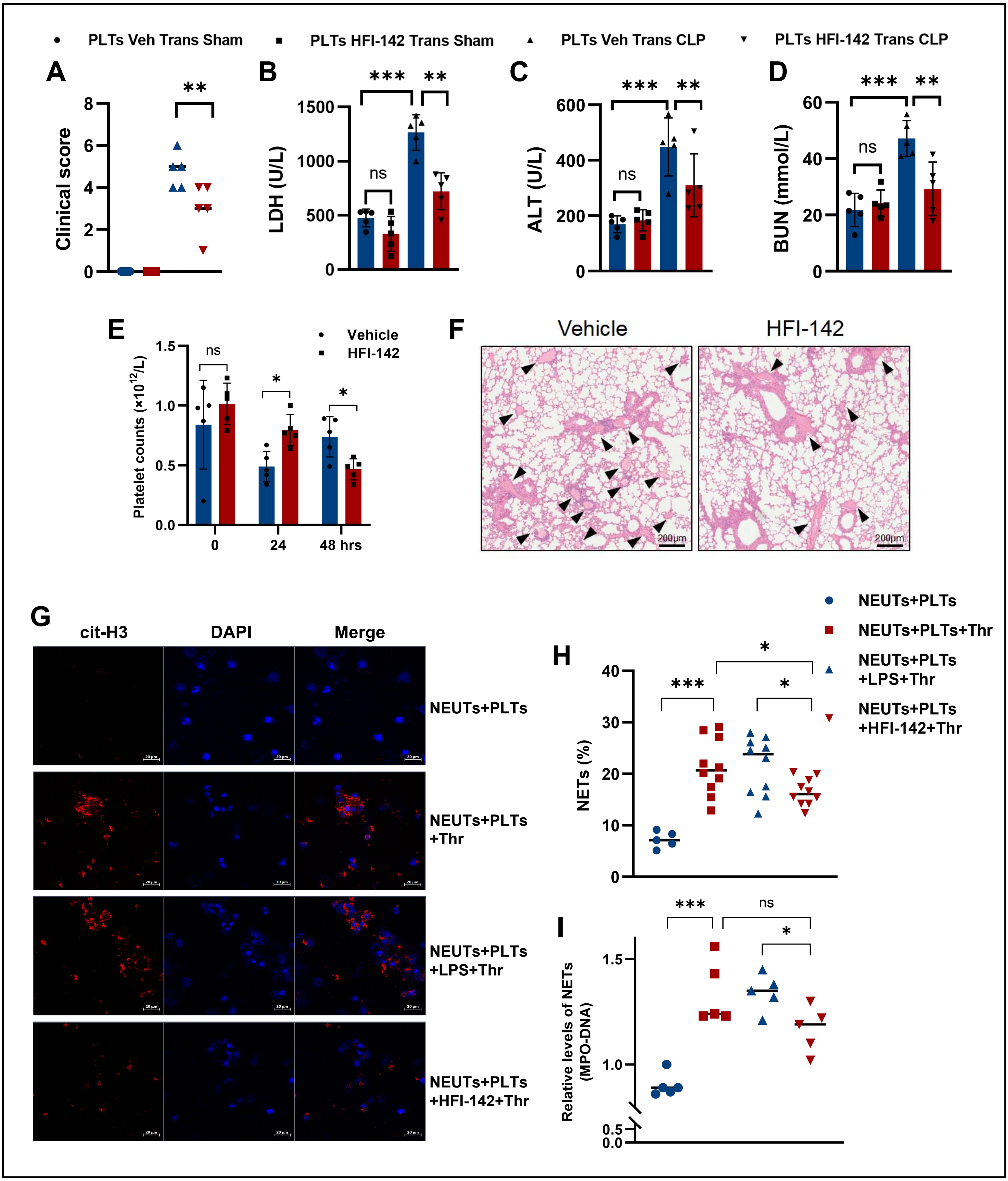
PLTs Veh Trans Sham
PLTs HFI-142 Trans Sham
PLTs Veh Trans CLP
PLTs HFI-142 Trans CLP
A
Clinical score
B
LDH (U/L)
C
ALT (U/L)
D
BUN (mmol/L)
E
Platelet counts (×10^12/L)
Vehicle
HFI-142
0
24
48 hrs
F
Vehicle
HFI-142
200μm
G
cit-H3
DAPI
Merge
NEUTs+PLTs
NEUTs+PLTs+Thr
NEUTs+PLTs+LPS+Thr
NEUTs+PLTs+HFI-142+Thr
H
NETs (%)
I
Relative levels of NETs (MPO-DNA)

Figure S7

Figure S7. Blocking IRAP S-acylation in platelets can alleviate septic symptoms and NETosis.

(A) Clinical score of sham and CLP-operated *Mpl*$^{-/-}$ mice transfused with platelets (treated with 0.9% NaCl or HFI-142).

(B–D) Blood serum biochemistry of lactate dehydrogenase (LDH) (B), liver enzyme alanine aminotransferase (ALT) (C) and blood urea nitrogen (BUN) (D) (n=5 in all groups) were measured in sham and CLP-operated *Mpl*$^{-/-}$ mice transfused with platelets (treated with 0.9% NaCl or HFI-142).

(E) Platelet counts in the peripheral blood of platelet-transfusion mice after CLP surgery at the indicated time points (n = 5).

(F) Hematoxylin-Eosin (HE) stained sections of lungs from CLP-operated platelet-transfusion *Mpl*$^{-/-}$ mice.

(G, H) Representative immunofluorescence images and analysis of NETs in vitro (isolated neutrophils cultured with platelets in the presence of LPS, Thrombin, HFI-142): DNA (blue) and cit-histone H3 (red). Scale bars, 20 μm.

(I) Quantitative analysis of NETs by MPO-DNA ELISA.

*p < .05, **p < .01, ***p < .001. ns, no significance; NEUTs, neutrophils; PLTs, platelets.
